# Isoprenylated Flavonoids and 2-Arylbenzofurans from the Root Bark of *Morus alba* L. and Their Cytotoxic Activity against HGC27 Cancer Cells

**DOI:** 10.3390/molecules29010030

**Published:** 2023-12-20

**Authors:** Hangyi Pu, Dongyi Cao, Xue Zhou, Fu Li, Lun Wang, Mingkui Wang

**Affiliations:** 1 Natural Products Research Center, Chengdu Institute of Biology, Chinese Academy of Sciences, Chengdu 610041, China; puhangyi18@mails.ucas.ac.cn (H.P.); zhouxue22@mails.ucas.ac.cn (X.Z.); lifu@cib.ac.cn (F.L.); wanglun@cib.ac.cn (L.W.); 2 Chengdu Institute of Biology, University of Chinese Academy of Sciences, Beijing 100049, China; 3 Pharmaceutical Department, The Third Affiliated Hospital of Yunnan University of Chinese Medicine, Kunming 650500, China; cdy1992126@126.com

**Keywords:** *Morus alba* L., gastric cancer, HGC27 cells, cytotoxic activities

## Abstract

Three new compounds (**1**, **11**, and **12**), together with 32 known ones, were isolated from the root bark of *Morus alba* L. using various chromatographic methods. The structures of the undescribed compounds were elucidated based on 1D, 2D NMR, and HRESIMS dataanalysis, while the known ones were identified by comparison of their spectroscopic data with those reported in the literature. All the isolates were evaluated for their cytotoxic activities against human gastric cancer HGC27 cells by CCK-8 assay. Among them, compounds **5**, **8**, **10**, and **30** exhibited cytotoxic activities on HGC27 cells with IC_50_ values of 33.76 ± 2.64 μM, 28.94 ± 0.72 μM, 6.08 ± 0.34 μM, and 10.24 ± 0.89 μM, respectively. Furthermore, compound **10** was confirmed to reduce proliferation ability, increase apoptosis rate, and inhibit cell migration pathway by annexin V/PI double staining experiment, transwell experiment, and Western blot analysis.

## 1. Introduction

*Morus alba* L., the root bark of *Morus* mulberry, is a well-known traditional Chinese medicine (TCM) documented in Chinese Pharmacopoeia [1]. The root bark of *M. alba* has been widely used in Chinese herbal medicinal prescriptions for the treatment of pneumonia, hypertension, diabetes, and arthritis [2,3]. Previous phytochemical studies revealed that *M. alba* contained a variety of polyphenolic constituents such as prenylated flavonoids, 2-arylbenzofurans, stilbene, coumarin, and mulberry Diels–Alder-type adducts. In addition, pharmacological studies exhibited that some polyphenols from *M. alba* had a broad spectrum of biological activities, such as hepatoprotective activity, whitening ability, and antiplatelet effects [4,5,6,7,8,9]. Previous studies also showed that diverse individual compounds or extracts from different parts of various mulberry plants (*M. alba*, *M. australis*, *M. wittiorum*, and *M. yunanensis*) exhibited anti-tumor activity against different tumor cell lines, such as lung cancer, liver cancer, colon cancer, breast cancer, rectal adenocarcinoma, and renal adenocarcinoma [10,11,12,13,14]. Our preliminary pharmacological experiment showed that the 75% ethanol extract of *M. alba* exhibited certain anti-tumor activity against gastric cancer cells, which had rarely been reported. Thus, the present paper focuses on detailed phytochemical investigation of the 75% ethanol extract of *M. alba* and evaluation of anti-tumor activity against gastric cancer cells of the isolated individual compounds. As a result, thirty-five compounds including three new ones were obtained. Among them, compound **10** (Albanol B) shows the most significant cytotoxicity with an IC_50_ value of 6.08 ± 0.34 μM. Moreover, the potential mechanism was preliminarily explored.

## 2. Results and Discussion

### 2.1. Isolation and Structure Elucidation

**Figure 1:**
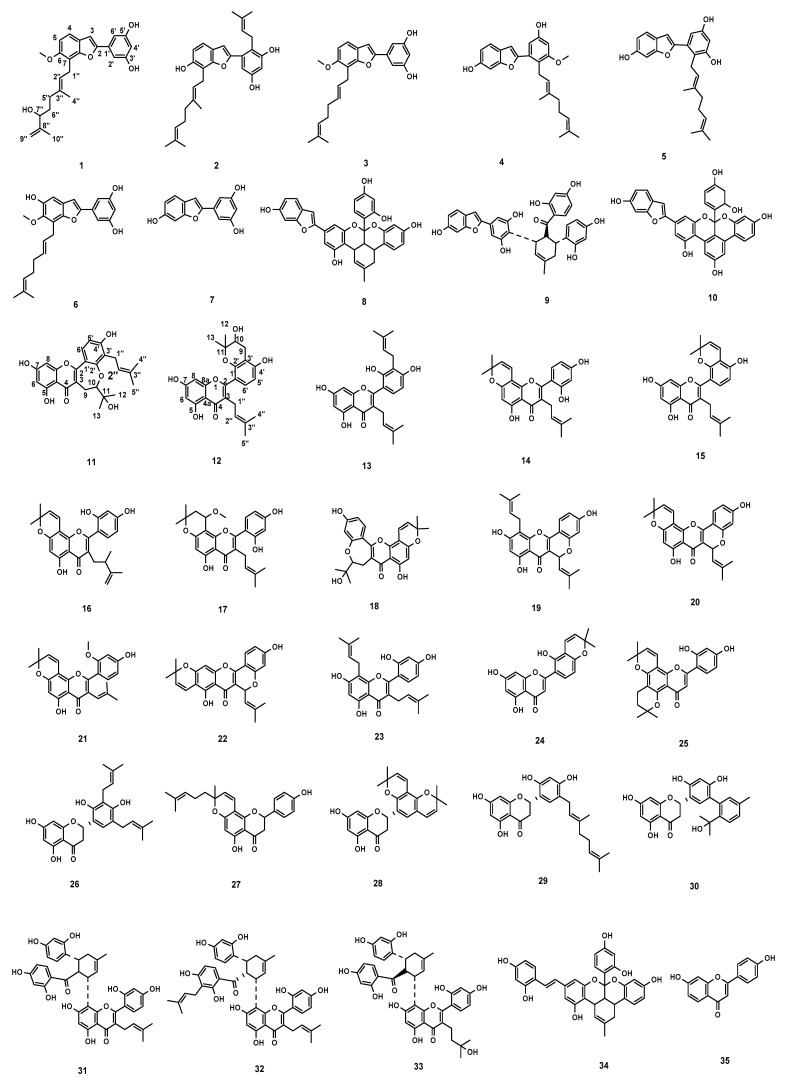
Chemical structures of compounds **1**–**35**.

Phytochemical investigation on the 75% ethanol extract of the root bark of *M. alba* led to the isolation and identification of 3 new compounds (**1**, **11**, and **12**) and 32 known compounds (**2**–**10**, **13**–**35**) as shown in Figure 1. The known compounds were identified by comparing their NMR and ESIMS data with those reported in the literature as mulberrofuran D (**2**) [15], mulberofuran B (**3**) [16], mulberofuran A (**4**) [17], albafuran A (**5**) [18], mulberrofuran Y (**6**) [19], moracin M (**7**) [20], mulberrofuran G (**8**) [21], mulberrofuran J (**9**) [22], abanol B (**10**) [23], kuwanon T (**13**) [21], morusin (**14**) [16], kuwanon A (**15**) [24], notabilisin L (**16**) [25], 14-methoxydihydromorusin (**17**) [26], neocyclomorusin (**18**) [27], cyclomulberrin (**19**) [28], cyclomorusin (**20**) [27], artoflavone (**21**) [29], cudraflavone A (**22**) [30], kuwanon C (**23**) [31], mornigrol H (**24**) [32], austraone A (**25**) [33], sanggenol Q (**26**) [34], cycloaltilisin 7 (**27**) [35], sanggenol O (**28**) [21], kuwanon E (**29**) [27], benzokuwanon E (**30**) [36], kuwanon G (**31**) [16], albanin G (**32**) [37], moracenin D (**33**) [38], mulberrofuran P (**34**) [39], and 7,4′-dihydroxyflavone (**35**) [40]. Among all compounds, **1**–**10** compounds are 2-arylbenzofurans and **11**–**35** compounds are isoprenylated flavonoids.

**Figure 2:**
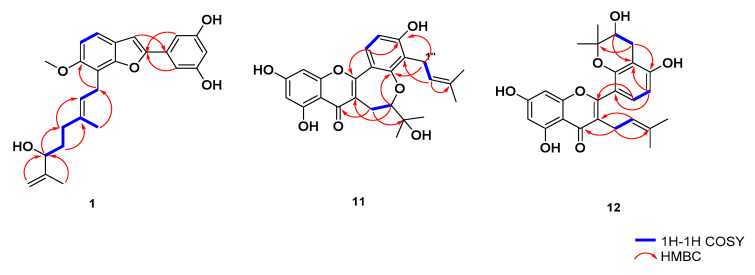
The ^1^H-^1^H COSY and key HMBC correlations of compounds **1, 11**, and **12**.

Compound **1** was obtained as a brown amorphous powder. The molecular formula of **1** was determined as C_25_H_28_O_5_ according to its HR-ESI-MS at *m*/*z* 438.1831 [M + Na]^+^ (calcd. for C_25_H_28_O_5_Na, 438.1829), indicating the presence of 12 degrees of unsaturation. Absorption maxima in the UV spectrum of **1** at *λ*_max_ 216 (4.21) and 316 (4.19) nm suggested that it was a 2-arylbenzofuran derivative [19]. In the ^1^H NMR spectrum (Table 1), three methyl groups at *δ*_H_ 1.62 (3H, s, H-10″), 1.89 (3H, s, H-4″), 3.86 (3H, s, -OCH_3_); four methylenes at *δ*_H_ 1.60 (2H, m, H-6″), 2.02 (2H, m, H-5″), 3.62 (2H, d, *J* = 7.2, H-1″), 4.79, 4.72 (2H, s, H-9″α, β); an olefinic singlet at *δ*_H_ 6.92 (1H, s, H-3), two aromatic doublet signals at *δ*_H_ 7.32 (1H, d, *J* = 8.4, H-4), *δ*_H_ 6.90 (1H, d, *J* = 8.4, H-5), a 1, 3, 5-trisubstituted aromatic system (ring B) at *δ*_H_ 6.80 (2H, d, *J* = 2.0, H-2′, 6′) and *δ*_H_ 6.26 (1H, s, H-4′). The ^13^C NMR (Table 1), DEPT and HSQC spectra showed 25 carbon signals, including three methyls, four methylenes, eight methines, and ten quaternary carbons. Comparison of the ^1^H and ^13^C-NMR spectral data of **1** with those of mulberofuran B (**3**) indicated that they shared similar chemical structures [18]. The marked difference occurred in the position of C-7″ of the geranyl group. The carbon signals for C-5″ and C-7″ were shifted downfield to *δ*_C_ 36.7 (−4.2 ppm) and 76.1 (−49.3 ppm), while the C-6″, C-8″, and C-9″ signals in **1** were shifted upfield dramatically to *δ*_C_ 34.2 (+6.5 ppm), 148.6 (+16.3 ppm), and 111.5 (+85.7 ppm) (Table 1), respectively. Signals in the upfield of the ^1^H NMR spectrum of **1** were deduced to be a changed geranyl group, one of whose double bonds was hydrated. Two proton signals at *δ*_H_ 4.72 (1H, s) and 4.79 (1H, s) and a signal at *δ*_H_ 3.90 (1H, t, *J* = 6.8 Hz) suggested that a terminal methylene group and an oxygen-bearing methine group were present in the geranyl group. According to the HSQC spectrum, δC 111.5 was connected with δH 3.90 (1H, t, *J* = 6.8 Hz), and δC 76.1 was connected to two protons with δH 4.79 (a, 1H, brs) and 4.72 (β, 1H, s). In the HMBC spectrum (Figure 2), long-range correlations of H-9″/C-7″, 10″ and H-7″/C-5″, 9″, 10″, demonstrated that the geranyl group was replaced by a 7″-hydroxy-3″, 8″-dimethylbut-2″, 8″dioctenyl group [41]. Furthermore, H-1″ showed long-range correlations with C-6 and C-7a, supporting that the changed geranyl group was located at C-7. According to the above NMR data analysis, compound **1** (shown in Figure 1) was elucidated as 7″-hydroxy-3″, 8″-dimethylbut-2″, 8″-dioctenyl-6-methoxy-3′, 5′-dihydroxy-2-arylbenzofuran, and was named mulberofuran Z.

Compound **11** was obtained as a yellow powder. The molecular formula of **11** was determined as C_25_H_26_O_7_ according to its HR-ESI-MS at *m*/*z* 439.1751 [M + H]^+^ (calcd. for C_25_H_27_O_7_, 439.1751), indicating 13 degrees of unsaturation. The IR spectrum exhibited absorption peaks attributable to hydroxy groups (3415 cm^−1^), a conjugated carbonyl group (1656 cm^−1^), and aromatic rings (1621, 1554, 1448 cm^−1^). In the ^1^H NMR spectrum (Table 2), signals due to an isopentyl group at *δ*_H_ 1.61 (3H, s, CH_3_-4″), 1.71 (3H, s, CH_3_-5″), 3.36 (2H, d, *J* = 6.0 Hz, H-1″), and 5.13 (1H, brt, *J* = 6.0 Hz, H-2″) were observed. The ^1^H NMR spectrum also showed ortho-coupled aromatic protons (ring B) at *δ*_H_ 7.67 (1H, d, *J* = 9.0 Hz, H-6′), 6.74 d (1H, d, *J* = 9.0 Hz, H-5′), and meta-coupled aromatic protons (ring A) at *δ*_H_ 6.12 (1H, brs, H-6), 6.33 (1H, brs, H-8). Two methyl groups *δ*_H_ 1.18 (3H, s, CH_3_-12), 1.29 (3H, s, CH_3_-13), and an AMX coupling system at *δ*_H_ 2.74 (1H, dd, *J* = 16.2, 6.0 Hz, H-9α), 3.00 (1H, dd, *J* = 16.2, 3.6 Hz, H-9β), and 3.99 (1H, dd, *J* = 6.0, 3.6 Hz, H-10) indicated the existence of an oxepin ring [42]. The ^13^C NMR (Table 2), DEPT, and HSQC spectra showed the presence of 25 carbons, including 4 methyls, 2 methylenes, 6 methines, and 13 quaternary carbons. Comparison of the ^1^H NMR and ^13^C NMR data of compound **11** with those of the known compound artoxanthocarpuone A [42] suggested that the difference between them was the location of the isopentyl group at C-3′ in **11** rather than at C-6 in artoxanthocarpuone A, which was further confirmed by the key long-range correlations from *δ*_H_ 3.36 (H-1″) to *δ*_C_ 159.4 (C-2′), 119.9 (C-3′), 156.4 (C-4′), and 130.3 (C-3″) (Figure 2). Based on these observations, compound **11** can be assigned as shown in Figure 1 and named artoxanthocarpuone C.

Compound **12** was obtained as a yellow powder. The molecular formula of **12** was determined as C_25_H_26_O_7_ according to its HR-ESI-MS at *m*/*z* 439.1749 [M + H]^+^ (calcd. for C_25_H_27_O_7_, 439.1751), indicating 13 degrees of unsaturation. The IR spectrum exhibited absorption peaks attributable to hydroxy groups (3372 cm^−1^), a conjugated carbonyl group (1645 cm^−1^). The UV spectrum showed absorbance maxima at 336 and 215 nm, typical of a flavone skeleton. The ^1^H NMR spectrum (Table 2) clearly exhibited the presence of an isopentyl group at *δ*_H_ 1.34 (3H, s, CH_3_-4″), 1.58 (3H, s, CH_3_-5″), 3.08 (2H, dd, *J* = 7.2, 5.4 Hz, H-1″), and 5.07 (1H, t, *J* = 7.2 Hz, H-2″). The ^1^H NMR spectrum also showed ortho-coupled aromatic protons (ring B) at *δ*_H_ 6.98 (1H, d, *J* = 8.4 Hz, H-6′), 6.42 (1H, d, *J* = 8.4 Hz, H-5′), and meta-coupled aromatic protons (ring A) at *δ*_H_ 5.91 (1H, s, H-6), 6.00 (1H, s, H-8). Two methyl groups *δ*_H_ 1.36 (3H, s, CH_3_-12), 1.28 (3H, s, CH_3_-13), and an AMX coupling system at *δ*_H_ 2.61 (1H, dd, *J* = 16.8, 5.4 Hz, H-9a), 2.95 (1H, dd, *J* = 16.8, 7.4 Hz, H-9b), and 3.80 (1H, dd, *J* = 16.2, 5.4 Hz, H-10) indicated the existence of an oxepin ring [42]. The ^1^H NMR and ^13^C NMR spectra (Table 2) of **12** were similar to those of the known compound kuwanon A [24]. The carbon signals at *δ*_C_ 70.2 (C-10) and 27.6 (C-9) in **12** instead of *δ*_C_ 128.9 (C-10) and 116.3 (C-9) in kuwanon A, along with the difference of their molecular weight, demonstrated the presence of a hydroxy group at C-10 in **12**. This was further verified by the long-range correlations from H-9 to C-11, C-1′ and C-4′ and from H-10 to C-11 and C-3′ in the HMBC spectrum (Figure 2) [43]. The HMBC correlations of H-1″/C-2, C-4 and H-2″/C-3, C-4″, C-5″ suggested the isopentyl group was attached to C-3 position. According to the above data, compound **12** was established as depicted in Figure 1 and named kuwanon Z.

### 2.2. Biological Activities of Compounds against HGC27 Cancer Cells

**Figure 3:**
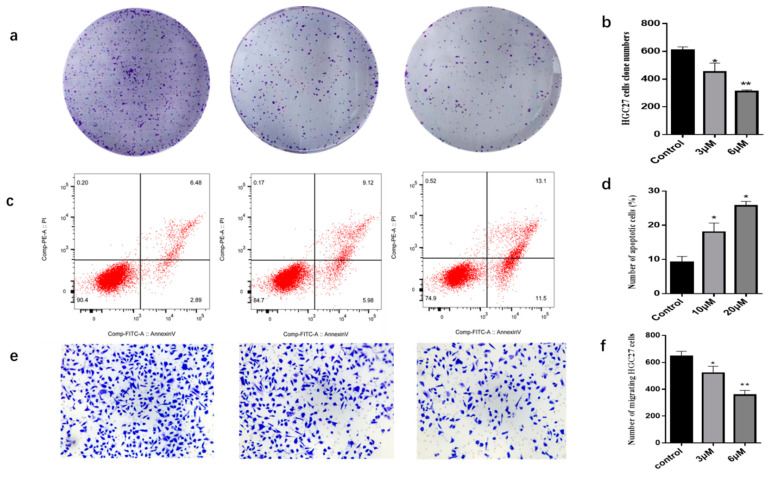
Cells apoptosis and migration assay. (**a**) Visual observation of cell clonogenicity. (**b**) Statistical analysis of cell clonogenicity. Data are mean ± SD (*n* = 3). Differences between treatment groups and control groups are determined, * *p* < 0.05, ** *p* < 0.01 as compared with control. (**c**) Apoptosis caused by **10** in HGC27 cells. (**d**) Treatment groups cells apoptosis rates were compared with control, * *p* < 0.05. Data are expressed as mean ± SD (*n* = 3). (**e**) The migration of HGC27 cells. (**f**) Quantitative evaluations of cell migration induced by **10** in the transwell assay, treatment groups were compared with control, * *p* < 0.05, ** *p* < 0.01. Data are expressed as mean ± SD (*n* = 3). Note: control: blank control group.

The effect of compounds (**1**–**35**) on the cell viability of HCG27 cells was evaluated by CCK-8 assay. As shown in Table 3, four compounds **5**, **8**, **10**, and **30** had a certain inhibitory effect on the viability of HCG27 cells compared with the model group (control). In contrast, other compounds had no significant inhibitory effects on the viability of HCG27 cells; therefore, compounds **5**, **8**, **10**, and **30** were screened to evaluate the semi-inhibition concentration (IC_50_) values (Table 4). Among the four compounds, compound **10** has showed the best cytotoxicity against HCG27 cells, with an IC_50_ value of 6.08 ± 0.34 μM, and compounds 5, 8, and 30 have showed certain cytotoxicity, with IC_50_ values of 33.76 ± 2.64 μM, 28.94 ± 0.72 μM, and 10.24 ± 0.89 μM, respectively. Compound **10** effectively inhibited the clone formation of HGC27 cells (Figure 3a). When cells were treated with 6 μM compound **10**, the number of cell clones formed in the administered group was significantly reduced and cell growth was significantly inhibited compared to the control group (Figure 3b). We used Annexin V-FITC (Annexin V-Fluorescein Isothiocyanate) double staining assay to detect apoptotic cells. As shown in Figure 3c, the apoptosis rate of HGC27 cells treated with compound **10** significantly increased compared to the control group. Quantification of apoptotic cells by flow cytometry revealed that the percentage of HGC27 apoptotic cells increased in a dose-dependent manner (Figure 3d).

**Figure 4:**
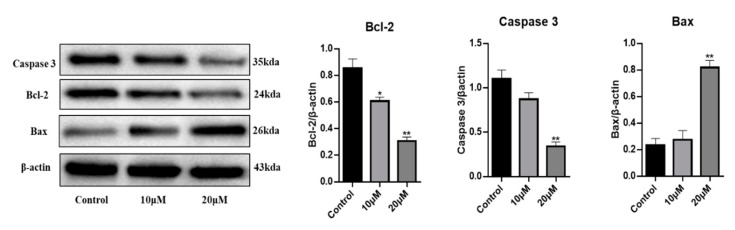
Compound **10** influences the expression of relevant proteins. Detection of Caspase-3, Bcl-2 and Bax proteins expression level in HGC27cells by Western Blot; the treatment groups were compared with control, * *p* < 0.05, ** *p* < 0.01. Data were expressed as mean ± SD (*n* = 3). Note: control: blank control group.

Cell migration is an essential process in tumor metastasis. The effect of compound **10** on migration was examined by the transwell method. The effect of compound **10** on the migration of HGC27 tumor cells was investigated in the premise of HGC27 cell death. As shown in Figure 3e, in vitro migration studies showed that compound **10** significantly inhibited the migratory ability of HGC27 cells in a dose-dependent manner. The number of migrating HGC27 cells was reduced from 647 to 357 compared to the control group (Figure 3f). Caspase-3 is an important protein in the regulation of cell apoptosis [44]. After treating HGC27 cells with 10 μM and 20 μM of compound **10** for 48 h, Western blot analysis showed the expression of apoptosis-related protein Caspase-3 was significantly increased compared with the control (Figure 4). Thus, compound **10** had a substantial cytotoxic effect on HGC27 cells (Figure 4). We also analyzed the expression levels of proteins Bax and Bcl-2 related to cell apoptosis [45]. Bax was upregulated in HGC27 cells, whereas Bcl-2 was downregulated. These results indicated that compound **10** has the capacity to induce apoptosis (Figure 4). 

## 3. Experimental

### 3.1. General Experimental Procedures

IR and UV spectra were recorded through a PerkinElmer 1725X-FT spectrometer with KBr disks and a PerkinElmer (Boston, MA, USA) Lambda 35 spectrometer, respectively, and optical rotations were determined by using a Perkin-Elmer 341 polarimeter. 1D and 2D NMR spectra were recorded using Bruker Avance 400 and 600 spectrometers. The HR-ESI-MS data were from micrOTOF-QII mass spectrometer. The precoated silica gel GF254 10–40 μm (Qingdao Marine Chemical Group, Qingdao, China) was used for TLC. Analytical HPLC was used with the Prominence LC-20AT with a model SPD-M20A detector and Ultimate ^®^C_18_ column (250 mm × 4.60 mm, 5 μm). Preparative HPLC was carried out on P3000 with a UV3000 detector (Chengdu LaiPu Science and Technology, Chengdu, China) and Ultimate ^®^C_18_ column (250 mm × 21.2 mm, 5 μm; 250 mm × 50 mm, 10 μm, respectively). Column chromatography (CC) was performed with silica gel (100–200 mesh and 200–300 mesh, Qingdao Marine Chemical Group, China) and RP-C18 (20–40 μm, Fuji, Japan) was used for column chromatography (CC). All chemical solvents were obtained from Chron Chemicals Reagent of Chengdu. The **35** compounds with more than 96% purity were prepared in our own laboratory.

### 3.2. Plant Material

The root bark of *M. alba* (Sang-Bai-Pi) was purchased from Lotus Pond Chinese Herbal Medicine market, Sichuan, China. It has been identified by Professor Weikai Bao (Chengdu Institute of Biology, Chinese Academy of Sciences). A voucher specimen (CIB-A-431) has been deposited at the Laboratory of Phytochemistry, Chengdu Institute of Biology, Chinese Academy of Sciences.

### 3.3. Extraction and Isolation

The air-dried and powdered roots (30.0 kg) were extracted three times with 75% ethanol (100 L × 3, each 3 days). The resulting solution was filtered, combined, and concentrated under reduced pressure to give 3.0 Kg of brown residue. The 75% ethanol extract (3.0 Kg) was partitioned between H_2_O and ethyl acetate. The ethyl acetate part (2.2 Kg) was separated by silica gel column (6.0 Kg, 150 × 40 cm) eluted with petroleum ether and ethyl acetate (10:1 to 1:1) to yield four fractions (Fr.1–4).

Part of Fr.1 (88.0 g) was separated by RP-HPLC (MeOH/H_2_O, 88:12) to afford seven fractions (Fr.1.1–Fr.1.7). Compound **13** (6.0 g) was obtained from Fr.1.2 (8.3 g) by crystallization from MeOH. Fr.1.3 (2.0 g) was fractionated with RP-HPLC (MeOH/H_2_O, 72:28) to give compounds **1** (22.0 mg)**, 26** (120.0 mg), **16** (16.0 mg), followed by Sephadex LH-20 (MeOH/H_2_O, 70:30 to 70:30) to yield **11** (8.0 mg), **12** (6.6 mg). Compound **15** (8.0 g) was from Fr.1.4 (12.2 g) by crystallization from MeOH, also. Compound **2** (310.0 mg) was obtained from Fr.1.5 (1.6 g) by RP-HPLC (CH_3_CN/H_2_O, 64:36). Compound **14** (22.4 g) was isolated from Fr.1.6 (28.9 g) by crystallization in MeOH. Fr.1.7 (503.0 mg) was fractionated using RP-HPLC with CH_3_CN/H_2_O (70:30) to give compounds **17** (18.0 mg), **3** (130.0 mg), **18** (30.0 mg), **19** (46.0 mg), **4** (22.0 mg), **20** (36.0 mg).

Part of Fr.2 (33.0 g) was also separated by RP-HPLC (MeOH/H_2_O, 86:14) to yield six subfractions (Fr.2.1–Fr.2.6). Fr.2.2 (3.8 g) and Fr.2.4 (1.3 g) were further purified by RP-HPLC eluted with CH_3_CN/H_2_O (70:30) to give compounds **28** (109.0 mg, CH_3_CN/H_2_O, 70:30) and **21** (28.0 mg MeOH/H_2_O, 89:11). Compounds **27** (17.0 mg) and **22** (100.0 mg) were obtained from Fr.2.5 (300.0 mg) and Fr.2.6 (653.0 mg) by RP-HPLC eluted with (MeOH-H_2_O, 90:10) and (MeOH-H_2_O, 84:16), respectively.

Part of Fr.3 (12.6 g) was subjected to preparative RP-HPLC MeOH/H_2_O (86:14) to obtain six fractions (Fr.3.1–Fr.3.7). Three compounds **31** (15.7 mg), **30** (8.0 mg), and **7** (33.0 mg) were separated from Fr.3.1 (817.0 mg) through RP-HPLC eluted with MeOH/H_2_O (73:17). Compounds **23** (86.0 mg) and **5** (42.0 mg) were purified from Fr.3.2 (310.0 mg) by RP-HPLC (CH_3_CN/H_2_O, 63:27). Fr.3.3 (120.0 mg) and Fr.3.4 (800.0 mg) were further purified by RP-HPLC eluted with CH_3_CN/H_2_O (60:40) and CH_3_CN/H_2_O (64:36) to afford compounds **24** (28.0 mg), **6** (98.0 mg). Compound **29** (37.0 mg) was obtained from Fr.3.5 (900.0 mg) through RP-HPLC (MeOH/H_2_O, 78:22).

Part of Fr.4 (36.0 g) was submitted to RP- HPLC (CH_3_CN/H_2_O, 64:36) to yield six subfractions (Fr.4.1–Fr.4.6). Compounds **33** (303.0 mg), **34** (23.0 mg), and **35** (6.0 mg) were separated from Fr.4.2 (13.6 g) by RP-HPLC eluted with CH_3_CN/H_2_O (63:37). Fr. 4.3 (800.0 mg) was further purified by RP-HPLC (CH_3_CN-H_2_O, 45:55) to obtain compound **9** (168.0 mg). Fr.4.5 (500.0 mg) and Fr.4.6 (200.0 mg) were separated by RP-HPLC eluted with CH_3_CN/H_2_O (52:48) and CH_3_CN/H_2_O (62:38) to yield compounds **8** (40.0 mg) and **32** (160.3 mg). Compounds **10** (128.0 mg) and **25** (36.0 mg) were isolated from Fr.4.7 (1.6 g) through RP-HPLC (MeOH/H_2_O, 74:26).

### 3.4. Spectroscopic Data

Mulberofuran Z (**1**), Brown amorphous powder, [α]_D_^20^ + 3.0 (c 0.22, MeOH); UV (MeOH) *λ*_max_ (log ε) 216 (4.21), 316 (4.19) nm; IR (KBr) *ν*_max_ 3423, 1615, 1490, 1362, 1266, 1155, 1084 cm^−1^; HR-ESI-MS at *m*/*z* 431.1831 [M + Na]^+^ (calcd. for C_25_H_28_O_5_Na, 431.1829); ^1^H and ^13^C NMR data, see Table 1.

Artoxanthocarpuone C (**11**), Yellow powder, [α] _D_^20^ + 3.7 (c 0.22, MeOH); UV (MeOH) *λ*max (log ε) 216 (3.21), 327 (4.36) nm; IR (KBr) *ν*_max_ 3415, 1656, 1621, 1554, 1448 cm^−1^; HRESIMS *m*/*z* 439.1751 [M + H]^+^ (calcd. for C_25_H_27_O_7_, 439.1751); ^1^H and ^13^C NMR data, see Table 2.

Kuwanon Z (**12**)**,** Yellow amorphous powder, [α] _D_^20^ + 10.6 (c 0.22, MeOH); UV (MeOH), *λ*max (log ε) 215 (2.61), 336 (3.18) nm; IR (KBr) *ν*_max_ 3372, 1645, 1616, 1440, 1299, 1167 cm^−1^; HRESIMS *m*/*z* 439.1751 [M + H]^+^ (calcd. for C_25_H_27_O_7_, 439.1749); ^1^H and ^13^C NMR data, see Table 2.

### 3.5. Cell Culture

HGC27 cells were purchased from American Type Culture Collection (ATCC). All cells grew adherently. HGC27 cells were cultured in RPMI 1640 media, and MRC-5 cells were cultured in a minimal essential medium (Gibco) (MA, USA). All media contained 10% endotoxin-free, heat-inactivated FBS (Gibco) and were maintained at 37 °C in a humidified atmosphere of 5% CO_2_. 

### 3.6. Cytotoxicity Assay 

All compounds were tested for cytotoxicity against HGC27 cell lines utilizing the CCK-8 method as previously reported [46]. HGC27 cells (4 × 10^3^/well) were seeded in 96-well plates for CCK-8 assays after treated with compound (**1**–**35**) 10 µM for 24 h. Then, 10 μL of CCK-8 reagent (biofrox Z6789D144) was added into each well, and incubated for 4 h at 37 °C. Finally, the optical density (OD) was then measured at 450 nm. Cells were treated with varying concentrations of compounds **5**, **8**, **10**, and **30** (1, 2.5, 5, 10, 20, 30, 40, and 60 µM) for 24 h. As in the above method, the IC_50_ values were calculated.

### 3.7. Colony Formation Assay 

Colony formation assay was performed as described previously. Briefly, 500 cells per well were seeded in 12-well plates and then incubated for 7 d. The colonies were fixed in methanol for 20 min and stained with 2% crystal violet for 20 min. Then, the stain solution was removed using tap water, and the cells were air-dried. Finally, the number of colonies was counted.

### 3.8. Cell Apoptosis Assay

HGC27 cells were cultured in a 6-well plate at a density of 700,000 cells/well and incubated in a cell incubator for 24 h. When HGC27 cells were in the logarithmic growth phase, HGC27 cells were treated with 10 μM or 20 μM compound **10** for 48 h. Then, cells were collected by trypsin digestion without EDTA and washed twice with cold PBS. An amount of 5 μL Annexin V/Alexa Fluor 488 solution was added to the cell suspension and incubated in the dark at room temperature for 5 min. Then, 10 μL PI and 400 μL PBS solution were added. The stained cell suspension was transferred to a flow tube, and the proportion of apoptotic cells was detected using BD Facscanto II, (Piscataway, NJ, USA), and the experimental results were statistically analyzed using Flowjo.

### 3.9. Transwell Migration Assay

Before the experiment, the cells were starved by culturing in a serum-free medium for 4 h. Then, cells were collected, and 30,000 cells were cells were treated with 3 μM and 6 μM Compound **10** inoculated into 500 μL serum-free medium in the upper chamber of Transwell (Corning, NY, USA), and 500 μL culture medium with 10% FBS was added into the lower chamber. After 24 h of incubation, the culture medium was discarded. Cells were fixed with 4% paraformaldehyde (Beyotime, Shanghai, China) for 15 min, then stained with 0.4% crystal violet (Beyotime, China) for 15 min. The cells were observed and photographed under a light microscope.

### 3.10. Western Blot Analysis

HGC27 cells which were treated with concentrations 10 µM and 20 µM compound 10 were lysed by RIPA lysis buffer (Beyotime, Shanghai) for 20 min. After centrifugation at 13,000 rpm for 25 min at 4 °C, the sediment was removed, and the supernate was mixed with 5 × Loading Buffer boiled for 5 min at 100 °C. Next, equal amounts of proteins were separated by sodium dodecyl sulfate-polyacrylamide gel electrophoresis (SDS-PAGE) and then transferred to polyvinylidene fluoride (PVDF) membranes (Millipore, Burlington, MA, USA). After blocked with 5% non-fat milk for 2 h at room temperature (RT), the membranes were incubated with the specific primary antibodies (Signalway Antibody, Nanjing, China) overnight at 4 °C. After washing three times with Tris Buffered saline Tween 20 (TBST), the membranes were then incubated with horseradish peroxidase (HRP)-conjugated secondary antibodies (ProteinTech, Wuhan, China) for 1 h at RT. The immune-blotting signals were detected using Beyo ECL Plus electrochemiluminescence reagent (Beyotime, Shanghai, China). Next, equal amounts of proteins were separated by sodium dodecyl sulfate-polyacrylamide gel electrophoresis (SDS-PAGE) and then transferred to polyvinylidene fluoride (PVDF) membranes (Millipore, USA).

## 4. Conclusions

Phytochemical investigation of the 75% ethanol extract of the root bark of *M. alba* led to the isolation and identification of three new compounds (**1**, **11**, and **12**) and 32 known ones (**2**–**10**, **13**–**35**). All compounds were tested for their cytotoxic activities against gastric cancer HGC27 cells. As a result, compounds **5**, **8**, and **30** exhibited certain cytotoxic activities with the IC_50_ values ranging from 33.76 ± 2.64 μM, 28.94 ± 0.72 μM, and 10.24 ± 0.89 μM, respectively. Compound **10** showed the most significant cytotoxic activity with an IC_50_ value of 6.08 ± 0.34 μM. Then, compound **10** was subjected to further experiments to explore its anti-tumor mechanism. It was found that compound **10** has inhibitory effects on proliferation and migration of human cancer HGC27 cells. In addition, the experimental results indicated that compound **10** was able to induce apoptosis through upregulation of Bax protein expression and downregulation of Caspase-3 and Bcl-2 protein expression. This study was the first time to preliminarily explore the mechanism of compound **10** in inhibiting gastric cancer cell HGC27 in vitro. In conclusion, the present study provides a reference for the phytochemical and biological activities of *Morus alba* L.

## Figures and Tables

**Table 1:** ^1^H NMR (400 MHz) and ^13^C NMR (100 MHz) data of **1** in CD3OD (*δ* in ppm, *J* in Hz).

No.	*δ*_H_ (*J* in Hz)	*δ*_C_	No.	*δ*_H_ (*J* in Hz)	*δ*_C_
2		156.7	1″	3.62 d (7.2)	23.6
3	6.92 s	102.3	2″	5.40 t (7.2)	123.6
3a		124.2	3″		135.9
4	7.32 d (8.4)	119.1	4″	1.89 s	16.4
5	6.90 d (8.8)	109.3	5″	2.02 m	36.7
6		156.4	6″	1.60 m	34.2
7		114.4	7″	3.90 t (6.8)	76.1
7a		155.3	8″		148.6
1′		133.8	9″α	4.79 s	111.5
2′	6.80 * d (2.0)	104.1	9″β	4.72 s	
3′		160.0	10″	1.62 s	17.5
4′	6.26 s	103.6	-OCH_3_	3.86 s	57.0
5′		160.0			
6′	6.80 * d (2.0)	104.1			

(*) refers to overlapped signals.

**Table 2:** ^1^H NMR and ^13^C NMR data for the compounds **11** and **12** (δ in ppm, *J* in Hz).

	11 ^a^		12 ^b^	
No.	*δ*_H_ (*J* in Hz)	*δ*_C_	*δ*_H_ (*J* in Hz)	*δ*_C_
2		158.6		163.1
3		115.7		122.1
4		179.7		183.1
4a		102.0		105.4
5		157.3		163.2
6	6.12 brs	98.9	5.91 ^a^ s	99.7 ^a^
7		161.3		165.8
8	6.33 brs	93.6	6.00 ^a^ s	94.0 ^a^
8a		157.3		157.1
9α	2.74 dd (16.2, 6.0)	23.0	2.61 dd (16.8, 5.4)	27.5
9β	3.00 dd (16.2, 3.6)		2.95 dd (16.8, 7.4)	
10	3.99 dd (6.0, 3.6)	91.5	3.80 dd (16.2, 6.0)	70.2
11		70.9		78.1
12	1.18 s	27.3	1.36 s	25.8
13	1.29 s	24.1	1.28 s	20.8
1′		114.0		113.8
2′		159.4		154.7
3′		119.9		110.3
4′		156.4		157.0
5′	6.74 d (9.0)	110.7	6.42 d (8.4)	109.9
6′	7.67 d (9.0)	126.8	6.98 d (8.4)	129.5
1″	3.36 *d (6.0)	22.5	3.08 dd (7.2, 5.4)	24.8
2″	5.13 t (7.2)	123.2	5.07 t (7.2)	122.6
3′		130.3		132.8
4″	1.61 s	25.5	1.34 s	17.6
5″	1.71.s	17.9	1.58 s	25.8
5-OH	13.02 s			

^a^ recorded at 600 (150) MHz in DMSO. ^b^ recorded at 600 (150) MHz in CD_3_OD. (*) refers to overlapped signals.

**Table 3:** Cell viability of compounds **1**–**35** on HGC27 cells.

Compound	Cell Viability (%)	Compound	Cell Viability (%)
**1**	96.42 ± 4.57	**19**	95.67 ± 3.65
**2**	97.88 ± 2.26	**20**	93.72 ± 2.86
**3**	96.27 ± 2.37	**21**	98.96 ± 0.92
**4**	92.08 ± 4.25	**22**	96.86 ± 1.84
**5**	67.31 ± 2.56	**23**	83.99 ± 3.92
**6**	89.04 ± 3.06	**24**	94.57 ± 2.74
**7**	89.47 ± 2.04	**25**	88.99 ± 4.62
**8**	59.92 ± 2.16	**26**	95.22 ± 3.30
**9**	96.62 ± 0.54	**27**	92.97 ± 2.72
**10**	39.71 ± 3.27	**28**	102.55 ± 3.06
**11**	96.27 ± 4.14	**29**	98.20 ± 0.37
**12**	74.89 ± 1.58	**30**	46.84 ± 3.02
**13**	77.66 ± 6.40	**31**	98.51 ± 0.97
**14**	95.05 ± 2.78	**32**	83.55 ± 1.51
**15**	97.90 ± 1.74	**33**	88.55 ± 3.20
**16**	97.01 ± 1.83	**34**	98.26 ± 1.03
**17**	98.36 ± 1.65	**35**	90.74 ± 5.24
**18**	95.05 ± 2.30	^a^ control	100.00 ± 1.31

^a^ control: blank control group. concentration: 10 µM.

**Table 4:** Inhibitory effects of compounds **5**, **8**, **10**, and **30** on HGC27 cells (mean ± SD, *n* = 3).

Compound	IC_50_ (µM)
**5**	33.76 ± 2.64
**8**	28.94 ± 0.72
**10**	6.08 ± 0.34
**30**	10.24 ± 0.89

## Data Availability

Data are contained within the article and supplementary materials.

## Supplementary Materials

The following supporting information can be downloaded at: https://www.mdpi.com/article/10.3390/molecules29010030/s1. Figures S1–S8, HR-ESI-MS data and NMR data of compound **1**, Figures S9–S15, HR-ESI-MS data and NMR data of compound **11**, Figures S16–S25, HR-ESI-MS data and NMR data of compound **12**.
