# Supplementary material for: Isoprenylated Flavonoids and 2-Arylbenzofurans from the Root Bark of Morus alba L. and Their Cytotoxic Activity against HGC27 Cancer Cells
Source: Molecules. 2023 Dec 20;29(1):30. doi: 10.3390/molecules29010030 (PMC10779849; doi:10.3390/molecules29010030)
Supplement: Supplementary file 1 [file molecules-29-00030-s001.zip › molecules-2745488-supplementary.pdf]

Isoprenylated Flavonoids and 2-arylbenzofurans from the Root Bark of *Morus alba* L. and Their Cytotoxic Activity against HGC27 Cancer Cells

Hang-Yi Pu [1,2], Dong-Yi Cao [3], Xue Zhou [1,2], Fu Li [1,2], Lun Wang [1,2] and Ming-Kui Wang [1,2,*]

[1] Natural Products Research Center, Chengdu Institute of Biology, Chinese Academy of Sciences, Chengdu 610041, China; puhangyi18@mails.ucas.ac.cn (H.-Y.P.); zhouxue22@mails.ucas.ac.cn (X.Z.); lifu@cib.ac.cn (F.L.); wanglun@cib.ac.cn (L.W.)
[2] Chengdu Institute of Biology, University of Chinese Academy of Sciences, Beijing 100049, China
[3] Pharmaceutical Department, The Third Affiliated Hospital of Yunnan University of Chinese Medicine, Kunming 650500, China; cdy1992126@126.com
* Correspondence: wangmk@cib.ac.cn; Tel. /Fax: +86-28-82890821

Supporting Information Available

Figure S1. HR-ESI-MS spectrum of compound **1**.

Figure S2. ^{1}H-NMR spectrum (400 MHz, CD_3OD) of compound **1**.

Figure S3. ^{13}C-NMR (100 MHz, CD_3OD) spectrum of compound **1**.

Figure S4. DEPT 135 spectrum (100 MHz, CD_3OD) of compound **1**.

Figure S5. HSQC spectrum of compound **1** in CD_3OD.

Figure S6. HMBC spectrum of compound **1** in CD_3OD.

Figure S7. ^{1}H-^{1}H COSY spectrum of compound **1** in CD_3OD.

Figure S8. NOSEY spectrum of compound **1** in CD_3OD.

Figure S9. HR-ESI-MS spectrum of compound **11**.

Figure S10. ^{1}H-NMR spectrum (600 MHz, DMSO) of compound **11**.

Figure S11. ^{13}C-NMR (150 MHz, DMSO) spectrum of compound **11**.

Figure S12. DEPT 135 spectrum (150 MHz, DMSO) of compound **11**.

Figure S13. HSQC spectrum of compound **11** in DMSO.

Figure S14. HMBC spectrum of compound **11** in DMSO.

Figure S15. ^{1}H-^{1}H COSY spectrum of compound **11** in DMSO.

Figure S16. HR-ESI-MS spectrum of compound **12**.

Figure S17. ^{1}H-NMR spectrum (600 MHz, CD_3OD) of compound **12**.

Figure S18. ^{13}C-NMR spectrum (150 MHz, CD_3OD) of compound **12**.

Figure S19. DEPT 135 spectrum (150 MHz, CD_3OD) of compound **12**.

Figure S20. HSQC spectrum of compound **12** in CD_3OD.

Figure S21. HMBC spectrum of compound **12** in CD_3OD.

Figure S22. ^{1}H-^{1}H COSY spectrum of compound **12** in CD_3OD.

Figure S23. ^{1}H-NMR spectrum (600 MHz, DMSO) of compound **12**.

Figure S24. ^{13}C-NMR (150 MHz, DMSO) spectrum of compound **12**.

Figure S25. DEPT 135 spectrum (150 MHz, DMSO) of compound **12**.

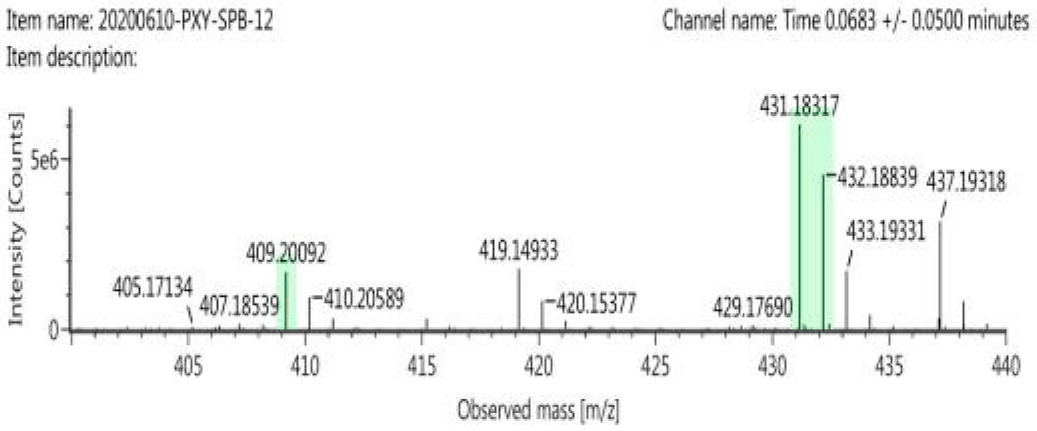

Figure S1. HR-ESI-MS spectrum of compound **1**.

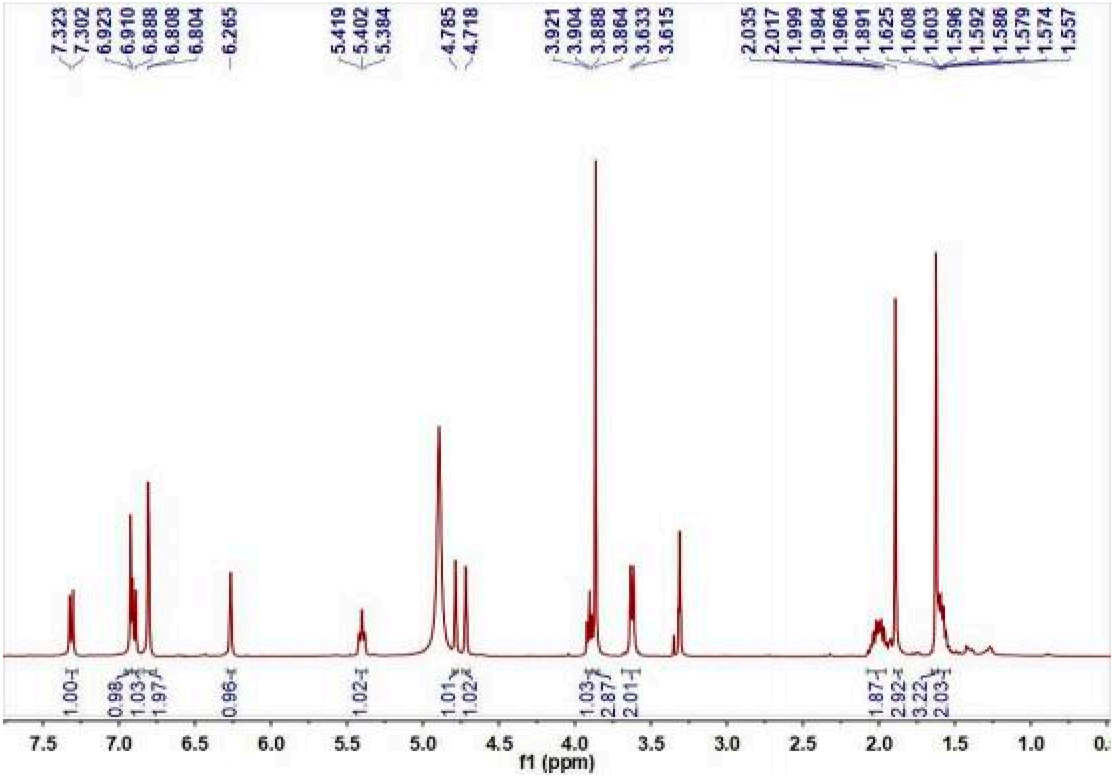

Figure S2. ^{1}H-NMR spectrum (400 MHz, CD_3OD) of compound **1**.

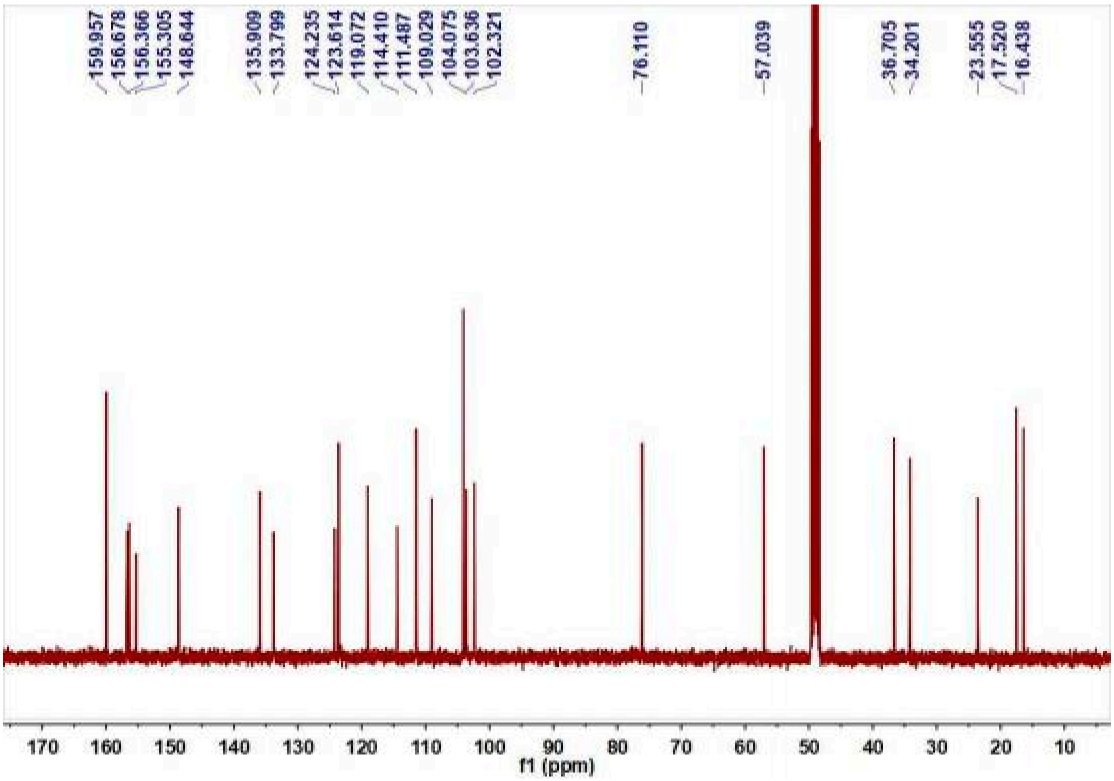

Figure S3. ^{13}C-NMR (100 MHz, CD_3OD) spectrum of compound **1**.

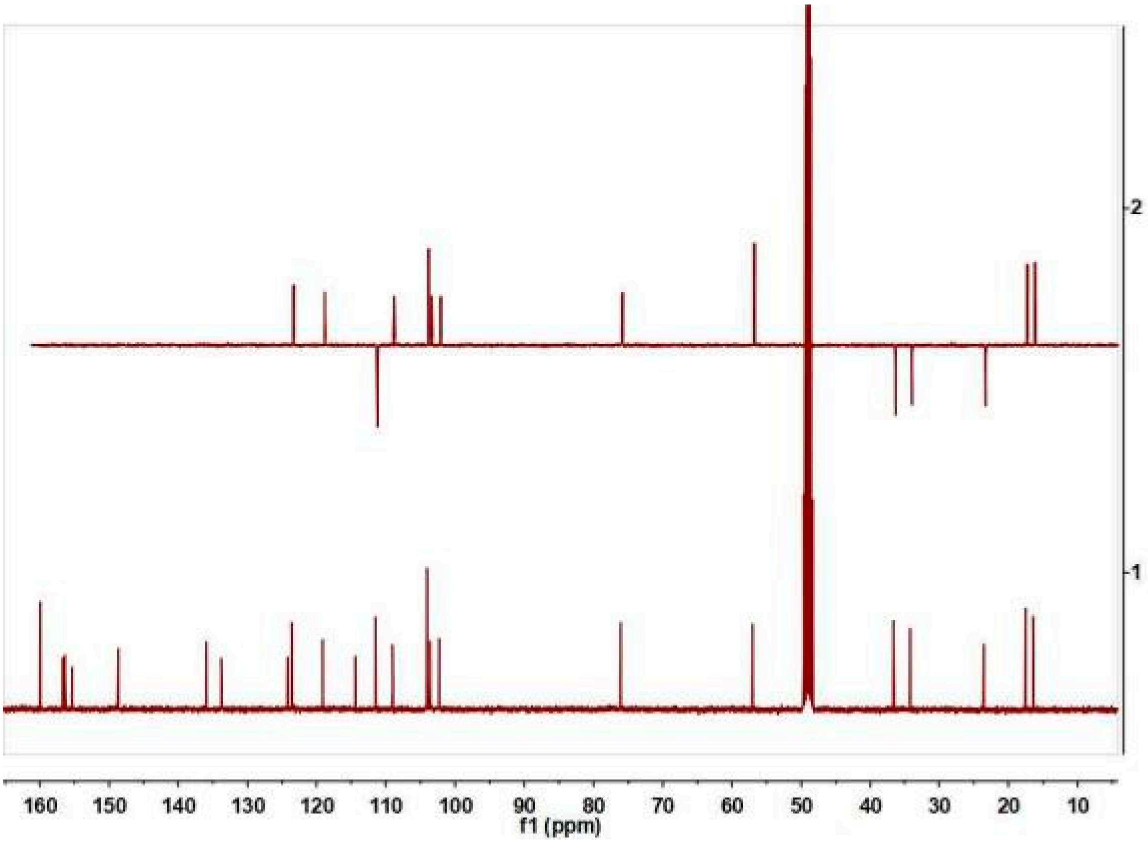

Figure S4. DEPT 135 spectrum (100 MHz, CD_3OD) of compound **1**.

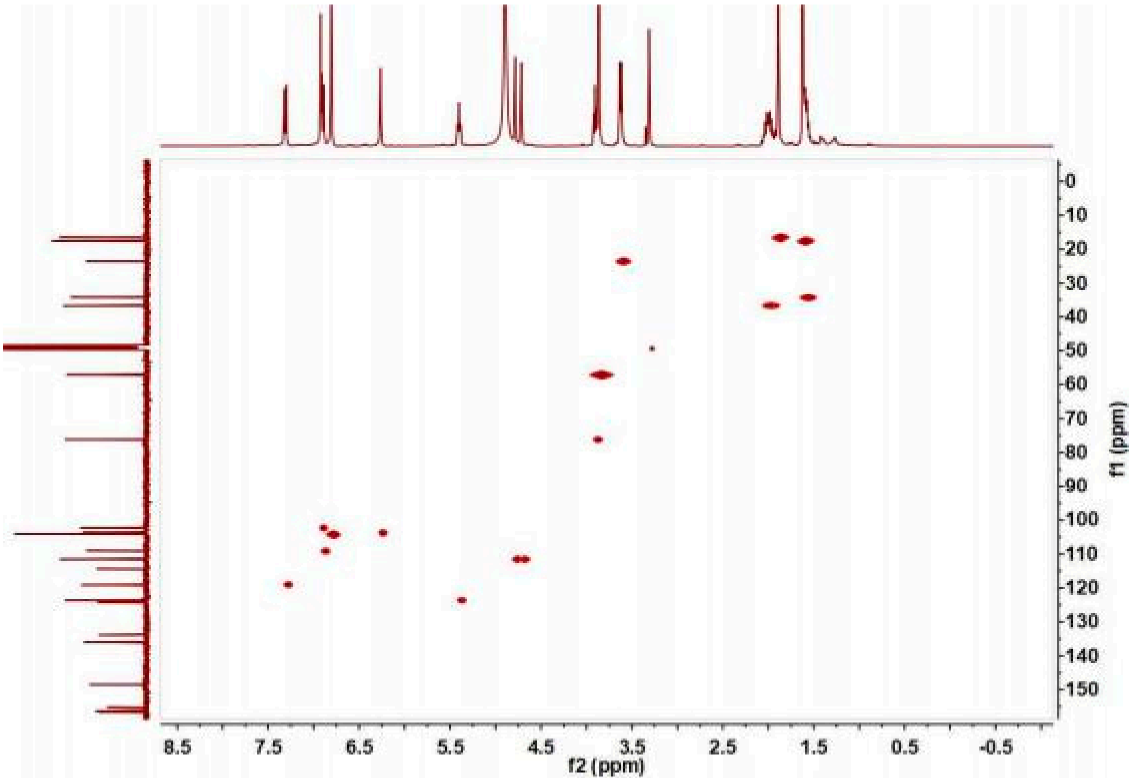

Figure S5. HSQC spectrum of compound **1** in CD_3OD.

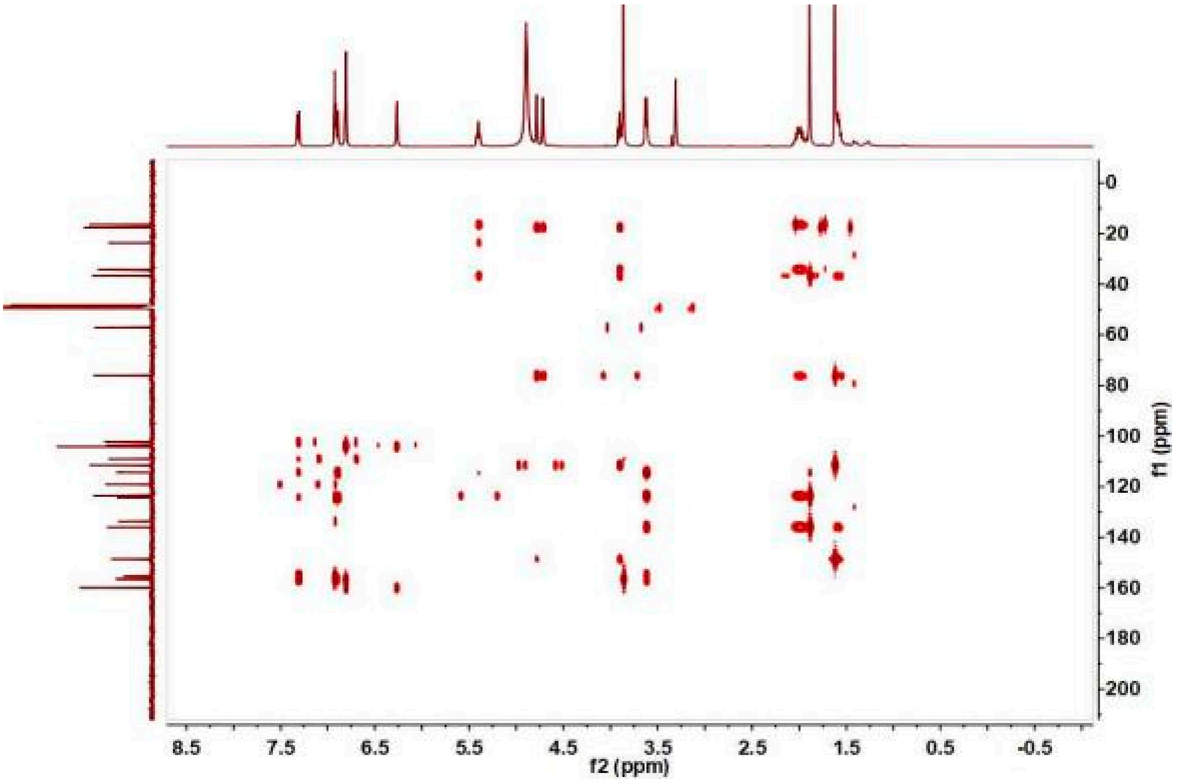

Figure S6. HMBC spectrum of compound **1** in CD_3OD.

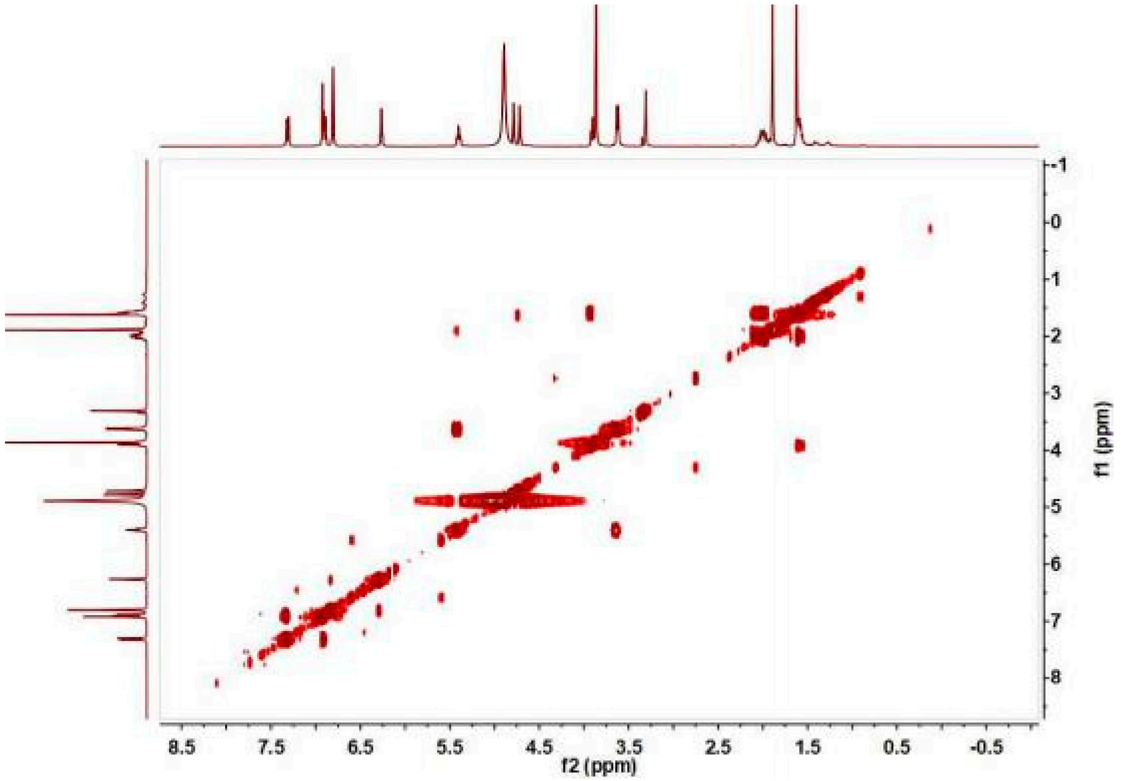

Figure S7. ^1H-^1H COSY spectrum of compound **1** in CD_3OD.

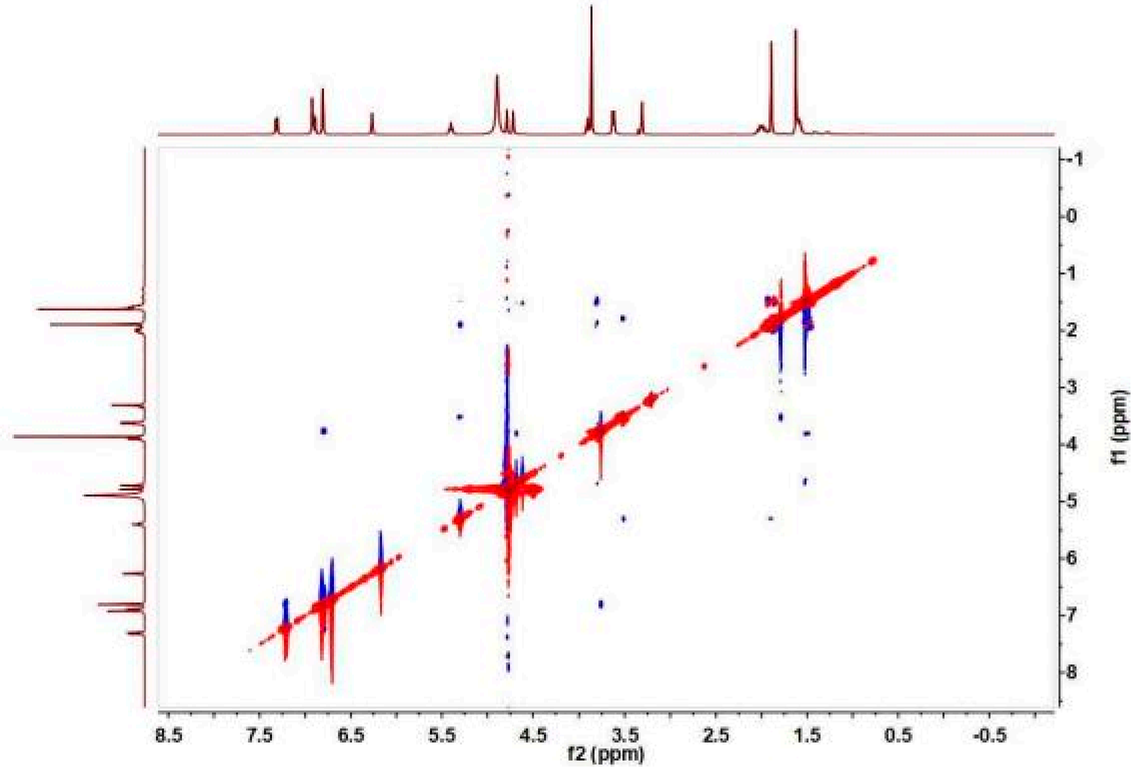

Figure S8. NOSEY spectrum of compound **1** in CD_3OD.

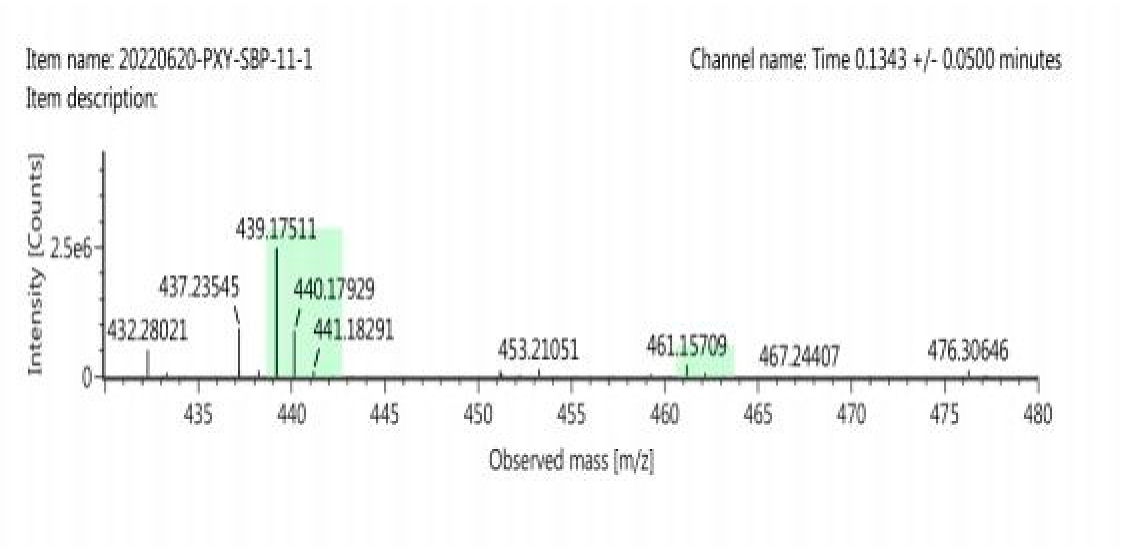

Figure S9. HR-ESI-MS spectrum of compound **11**.

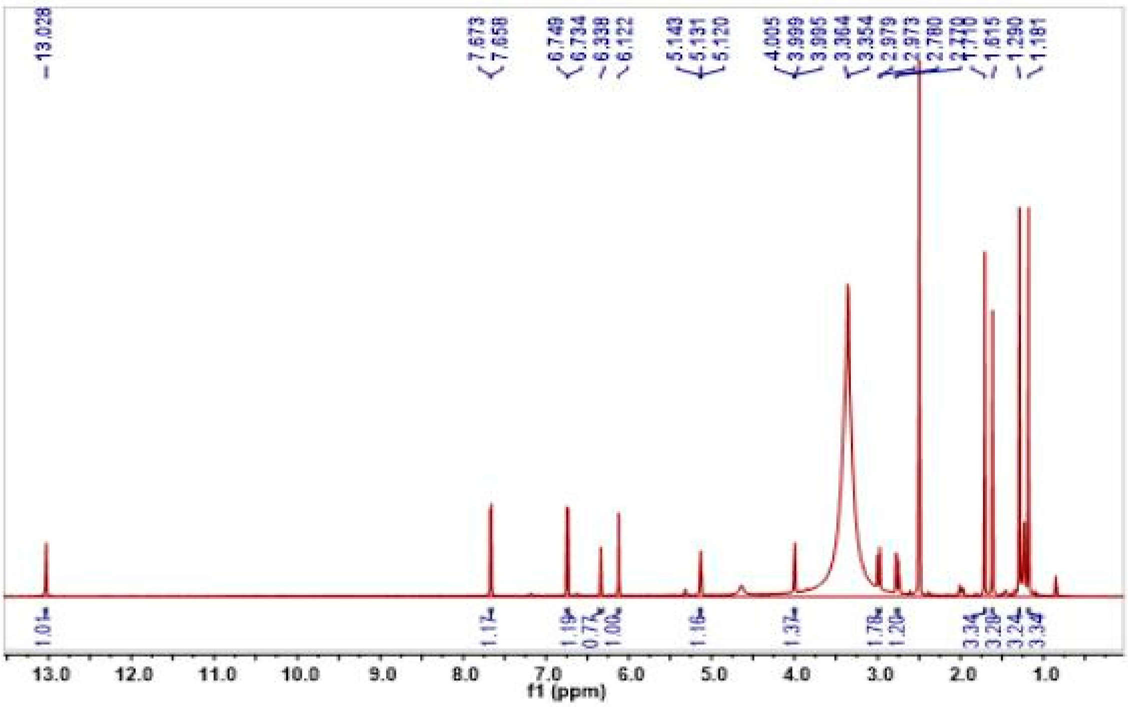

Figure S10. ^{1}H-NMR spectrum (600 MHz, DMSO) of compound **11**.

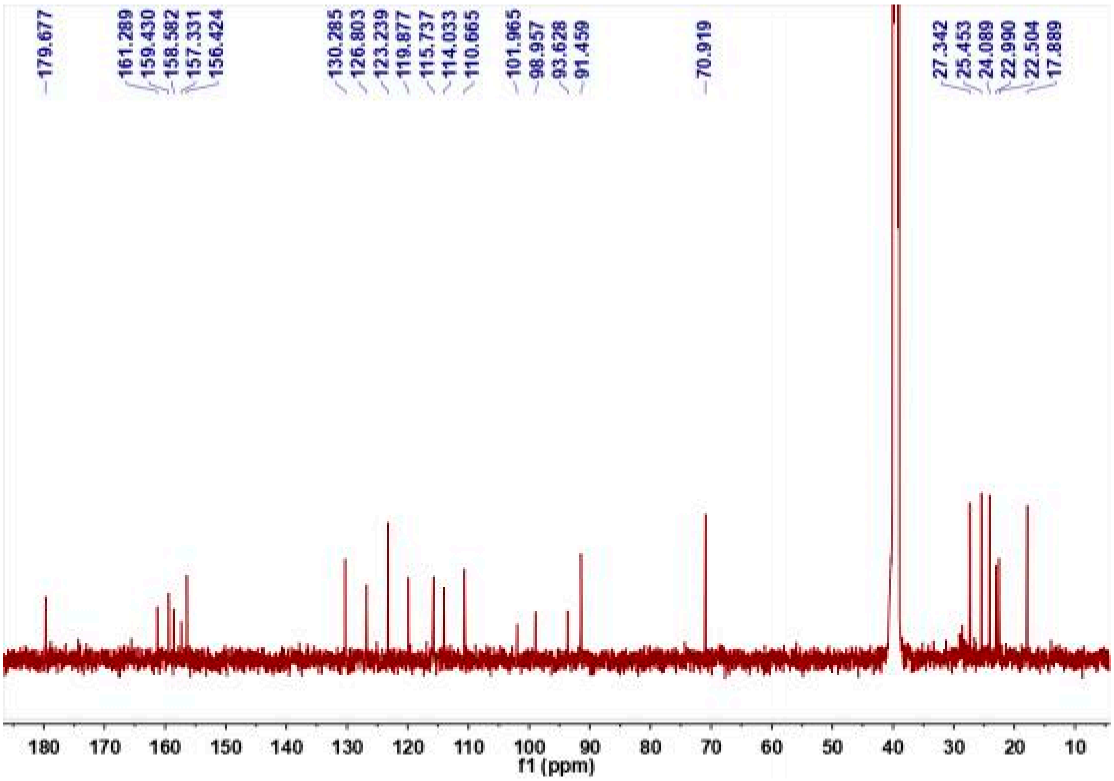

Figure S11. ^{13}C-NMR (150 MHz, DMSO) spectrum of compound **11**.

.

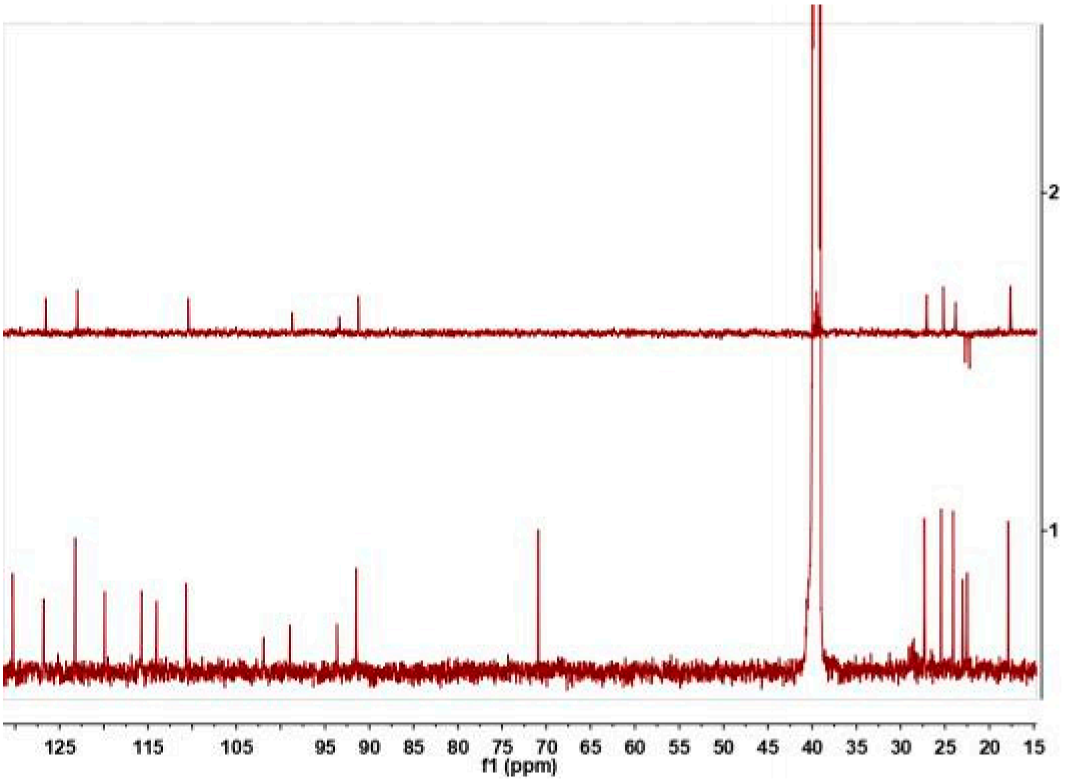

Figure S12. DEPT 135 spectrum (150 MHz, DMSO) of compound **11**.

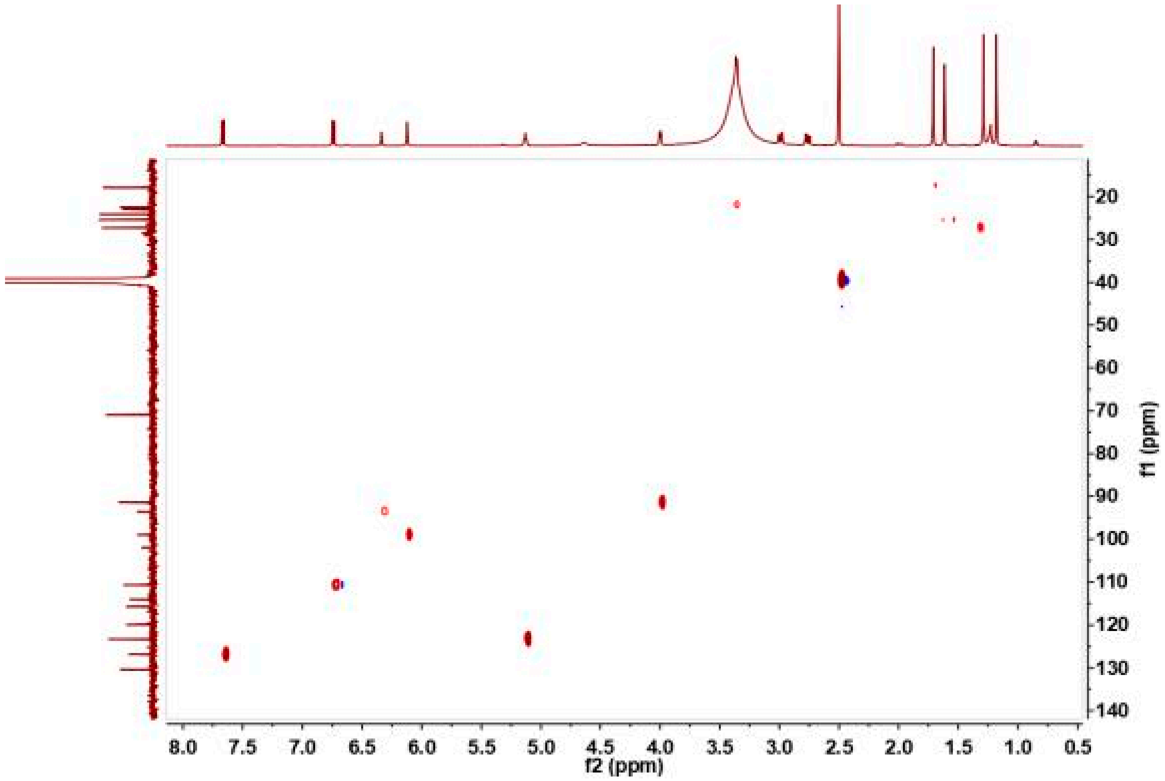

Figure S13. HSQC spectrum of compound **11** in DMSO.

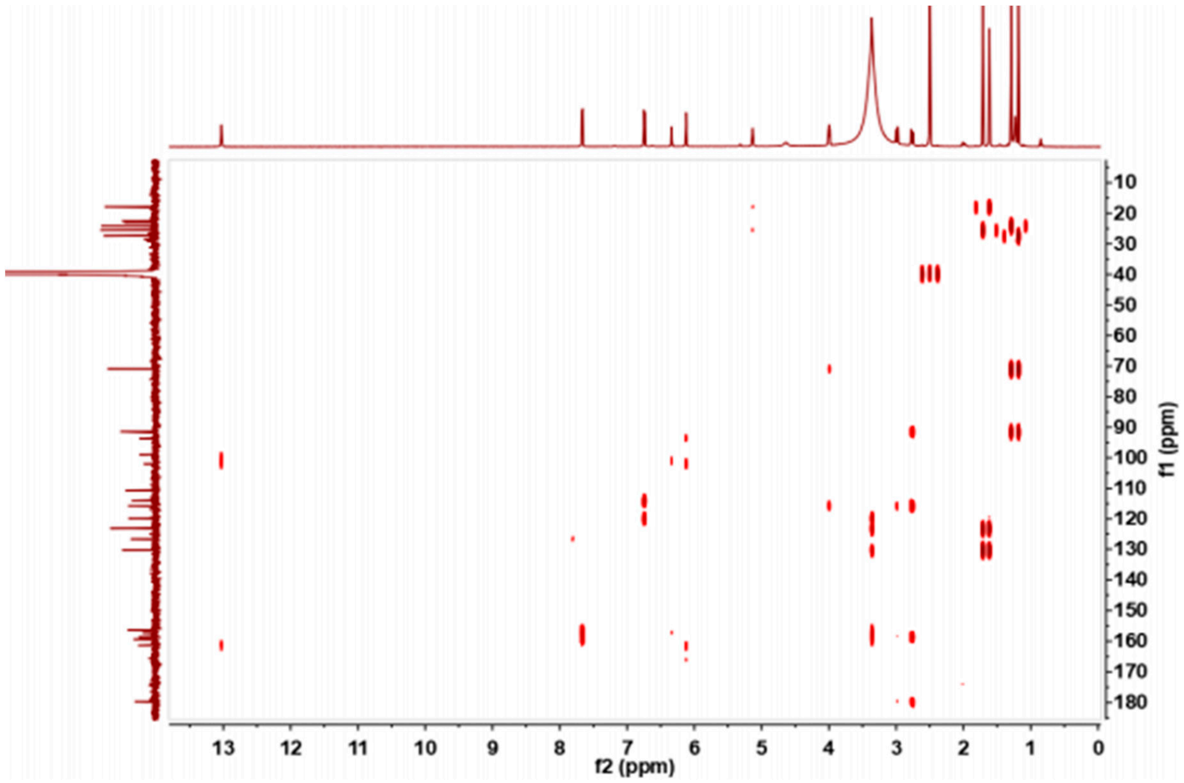

Figure S14. HMBC spectrum of compound **11** in DMSO.

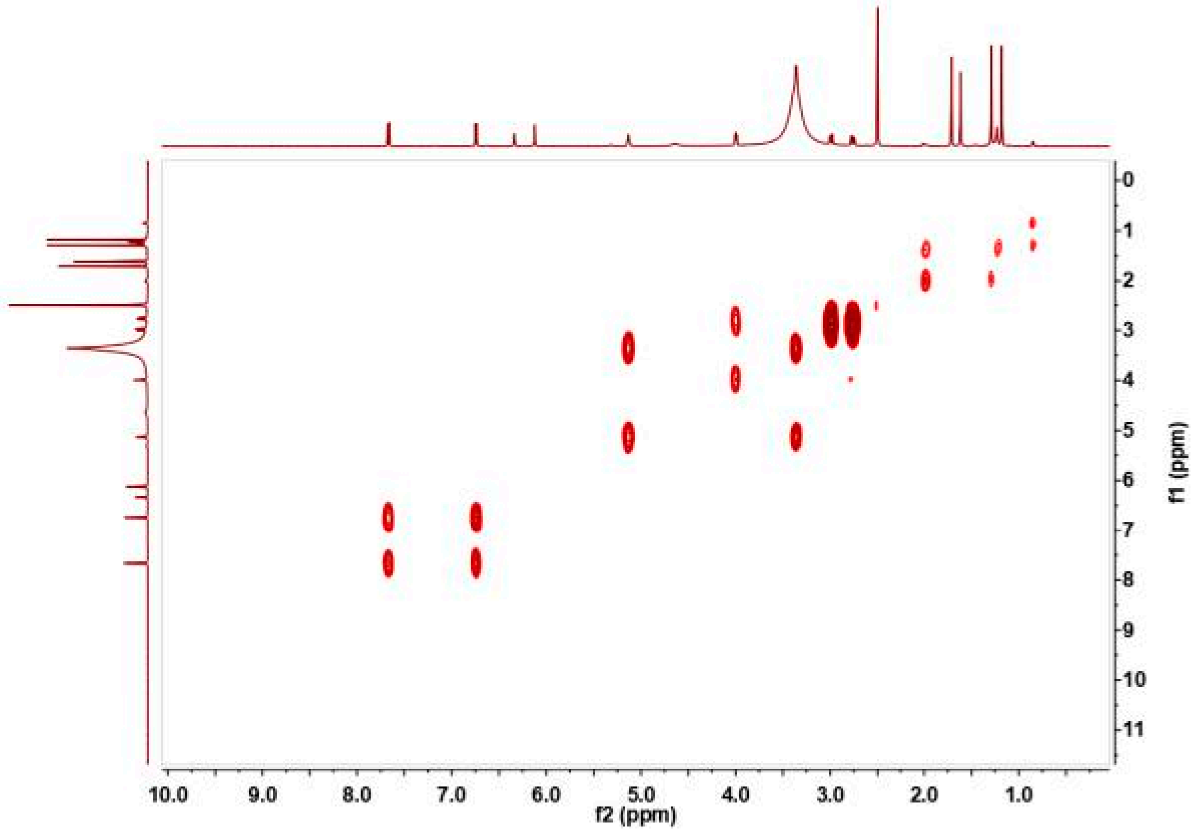

Figure S15. ^{1}H-^{1}H COSY spectrum of compound **11** in DMSO.

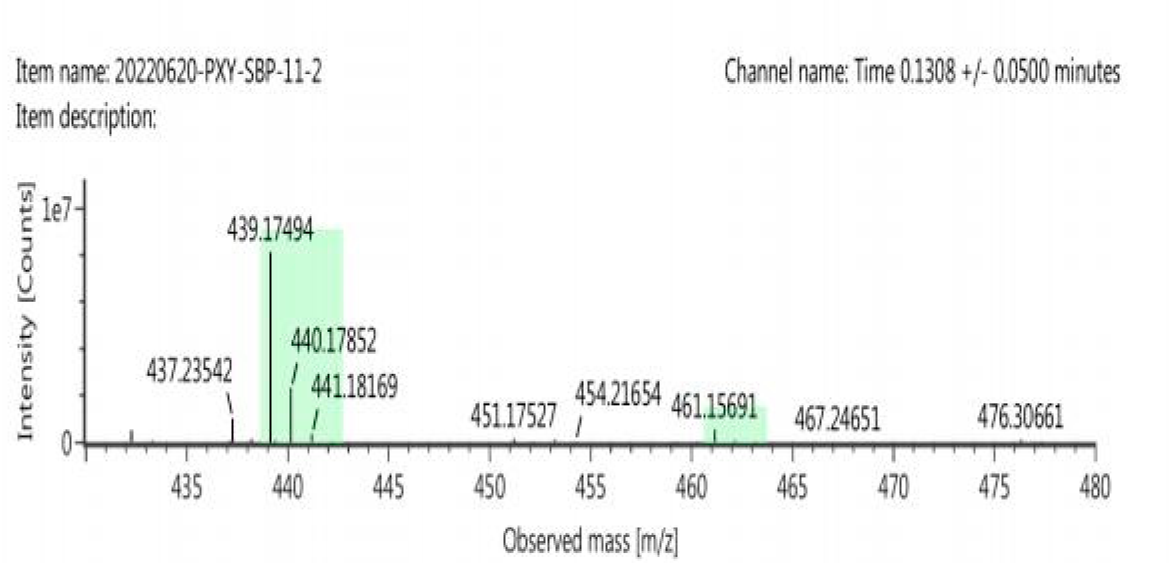

Figure S16. HR-ESI-MS spectrum of compound **12**.

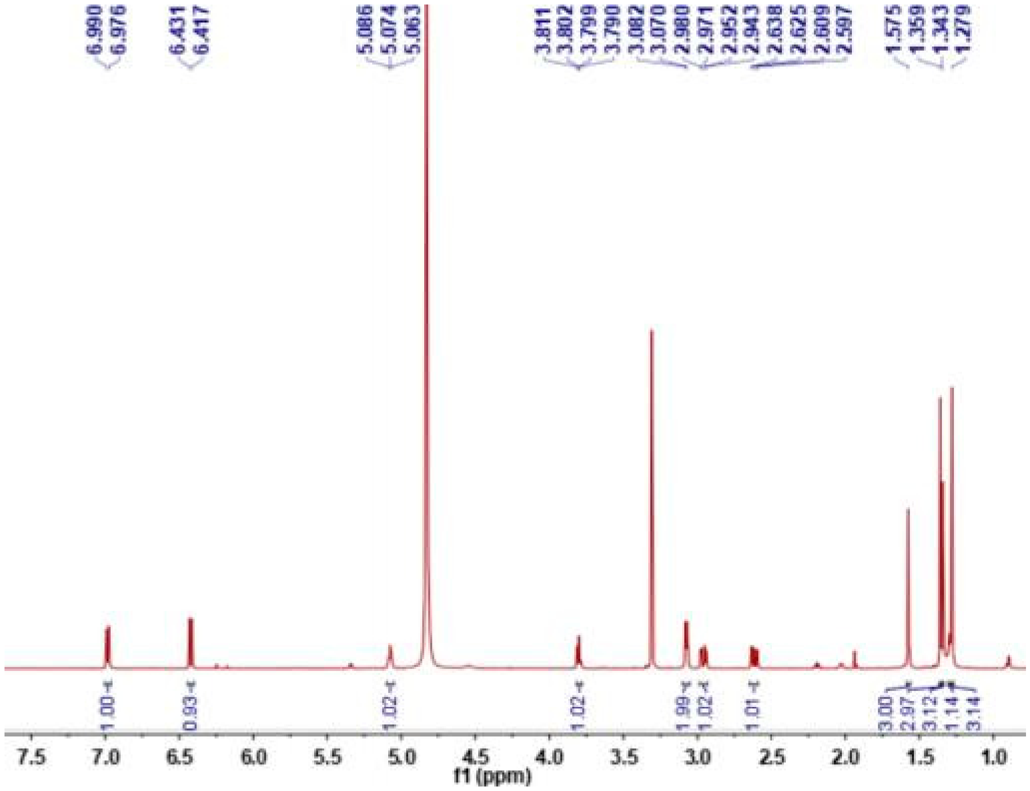

Figure S17. ^{1}H-NMR spectrum (600 MHz, CD_3OD) of compound **12**.

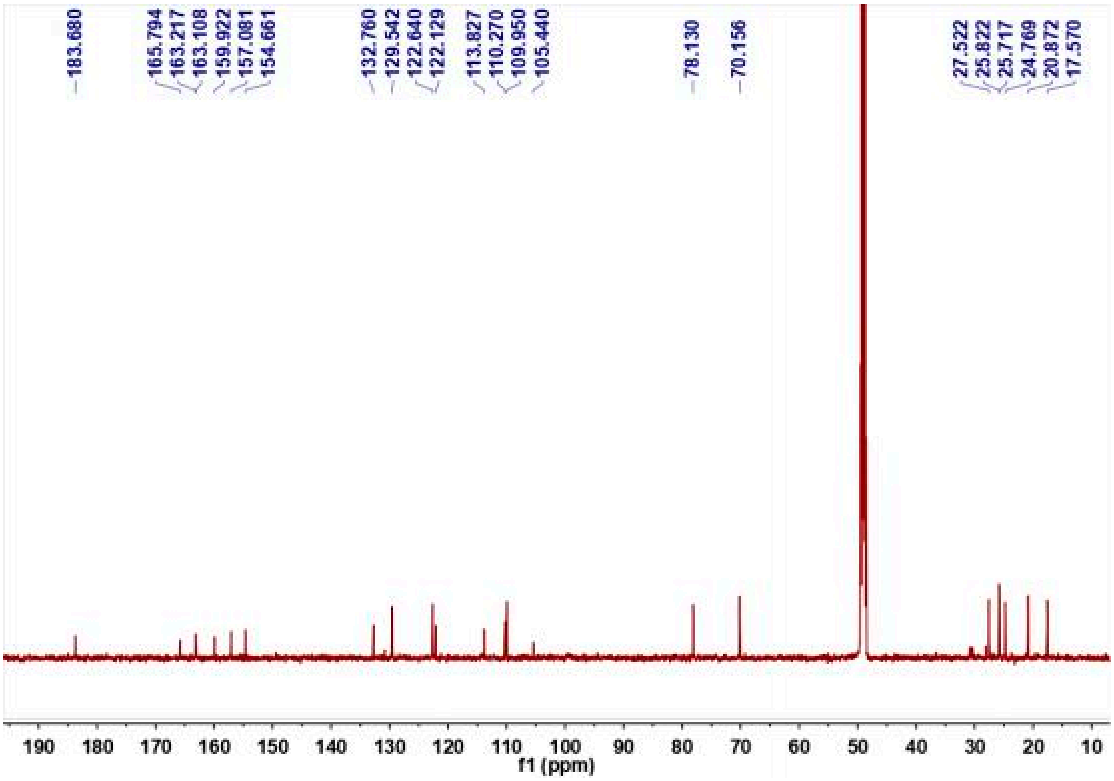

Figure S18. ^{13}C-NMR spectrum (150 MHz, CD_3OD) of compound **12**.

.

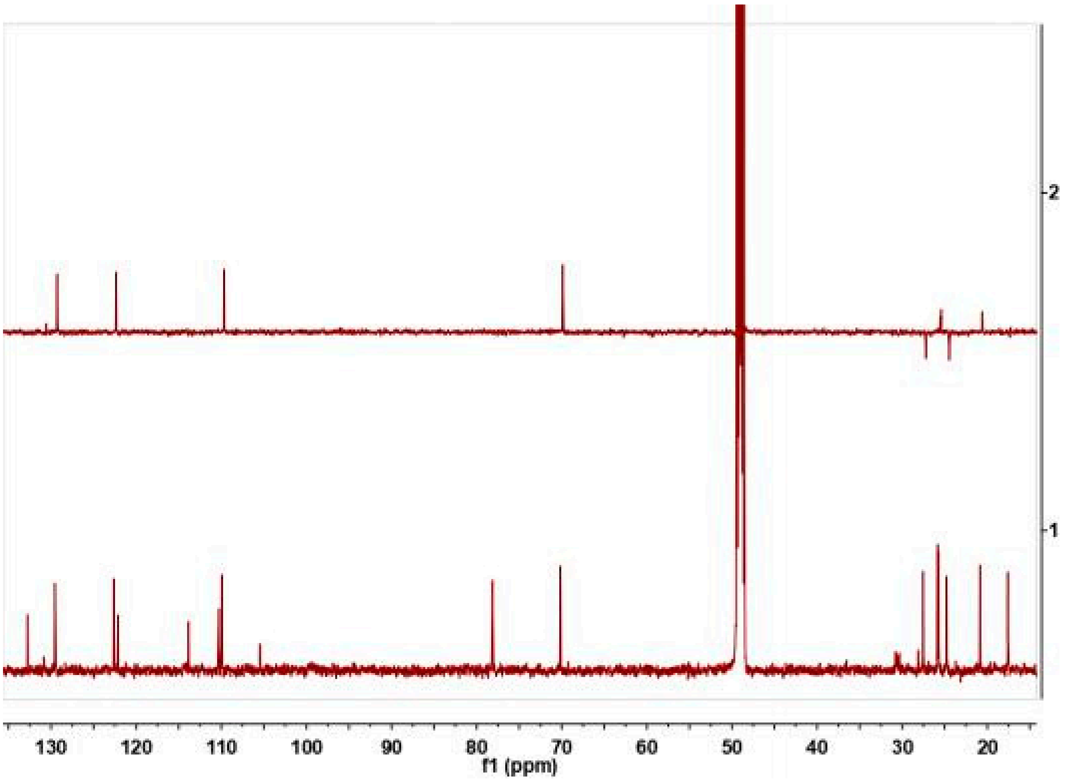

Figure S19. DEPT 135 spectrum (150 MHz, CD_3OD) of compound **12**.

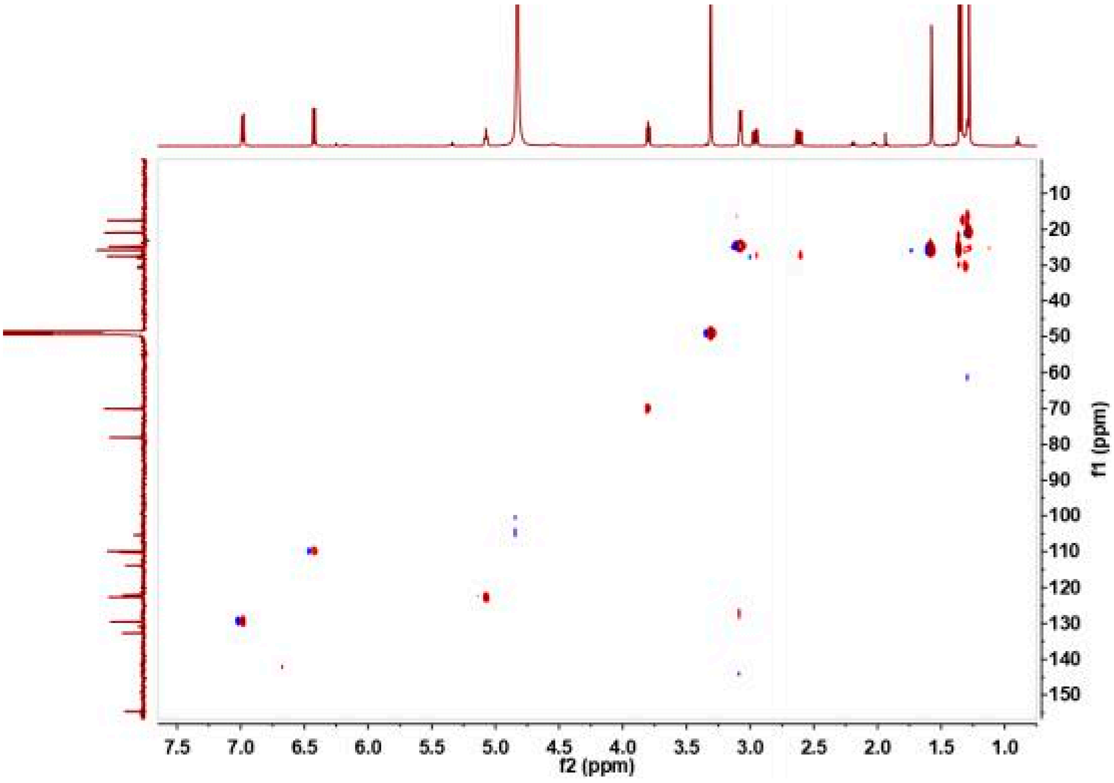

Figure S20. HSQC spectrum of compound **12** in CD_3OD.

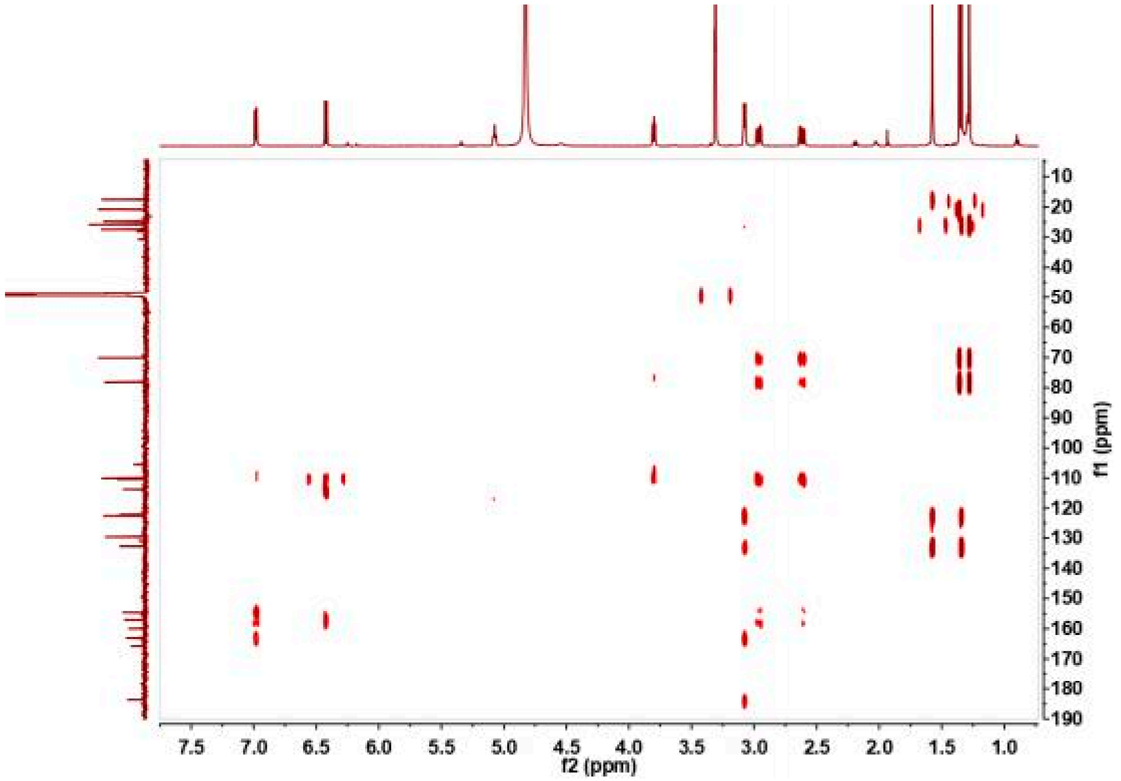

Figure S21. HMBC spectrum of compound **12** in CD_3OD.

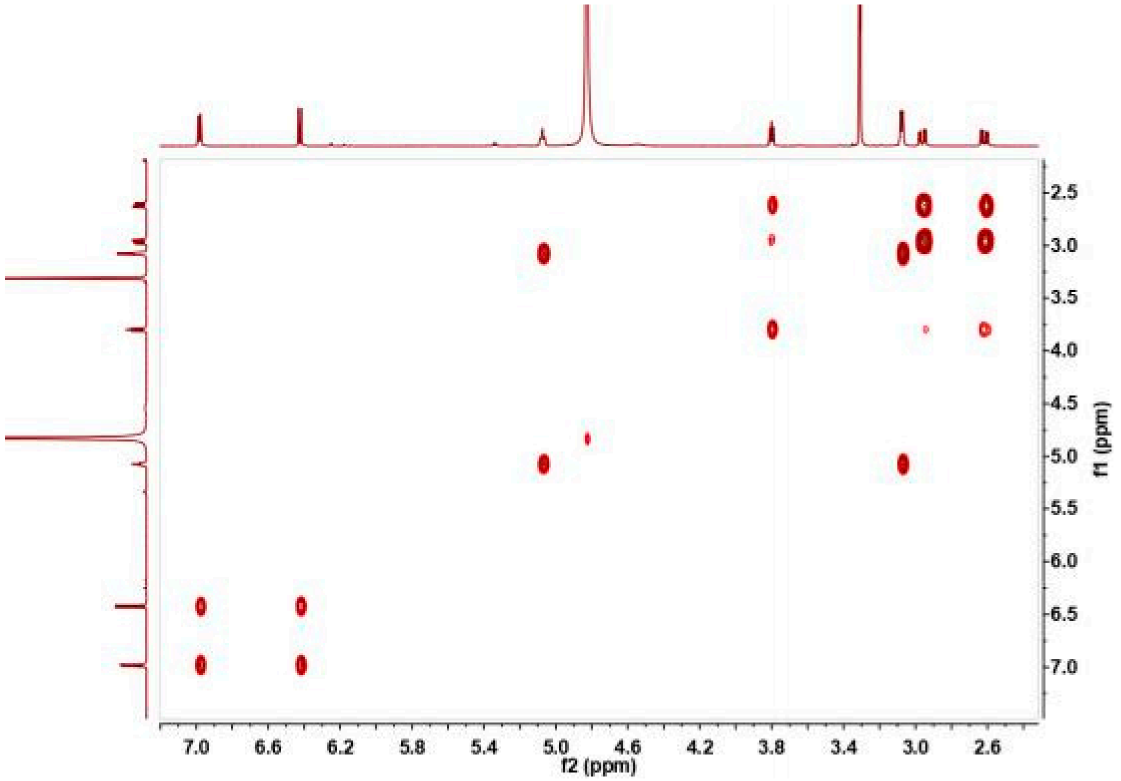

Figure S22. ^{1}H-^{1}H COSY spectrum of compound **12** in CD_3OD.

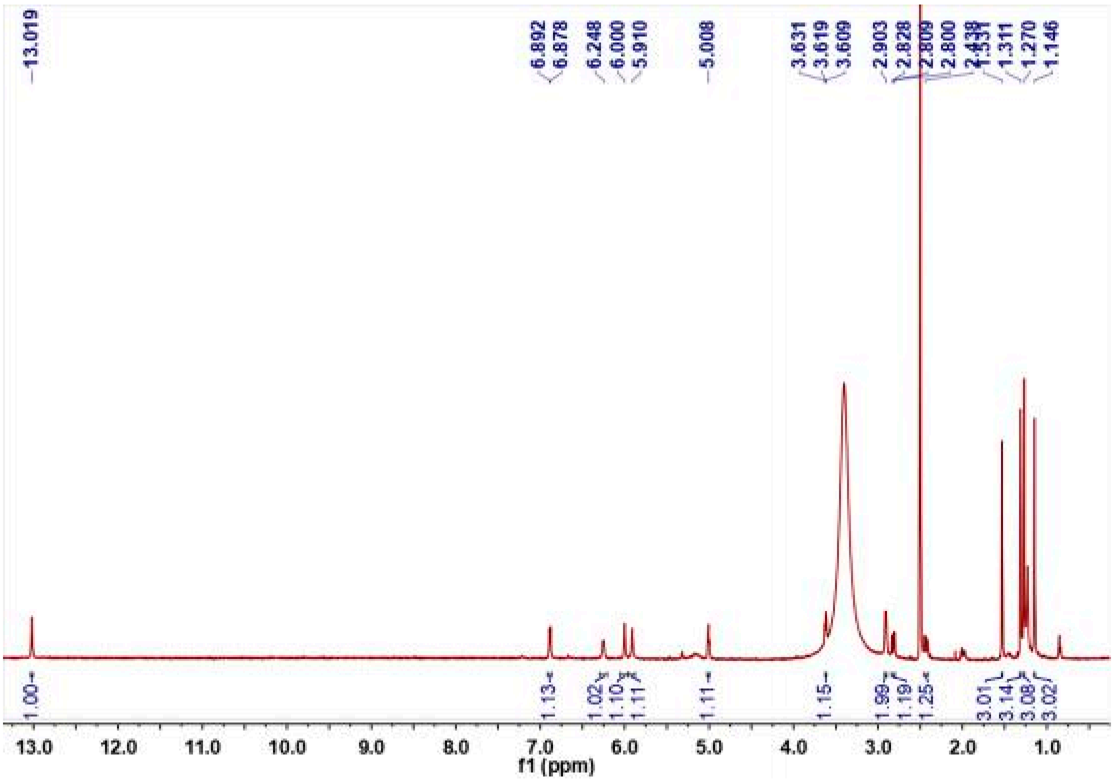

Figure S23. ^{1}H-NMR spectrum (600 MHz, DMSO) of compound **12**.

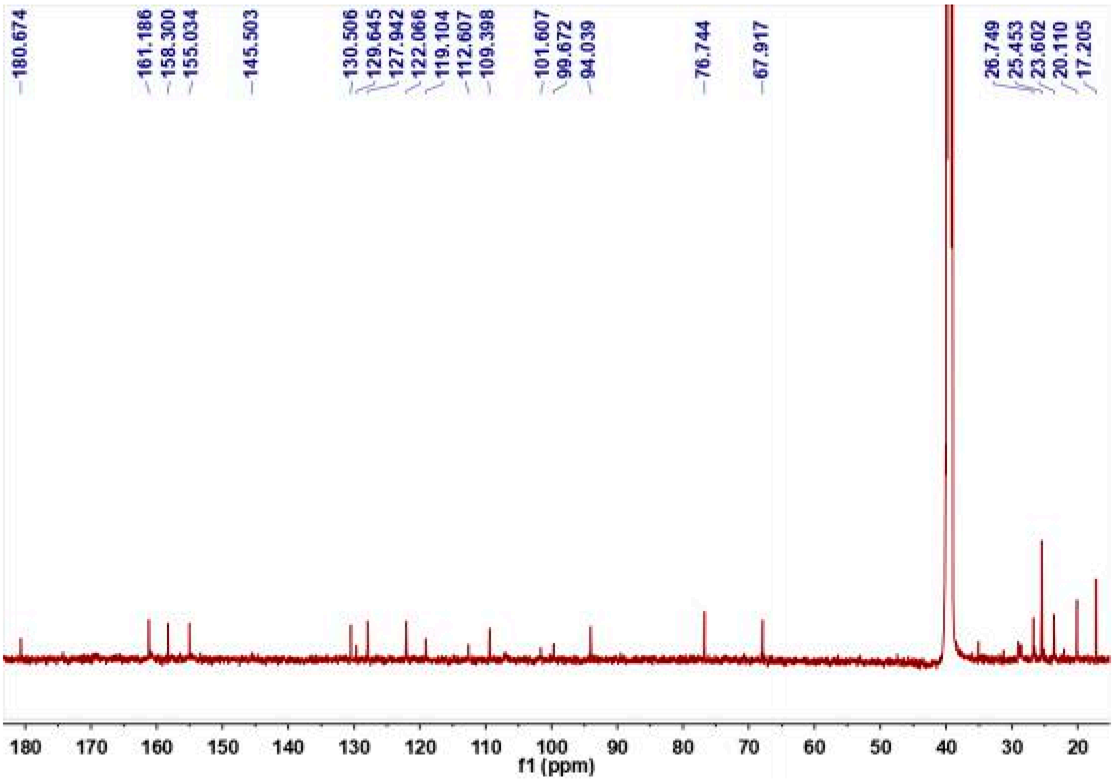

Figure S24. ^{13}C-NMR (150 MHz, DMSO) spectrum of compound **12**.

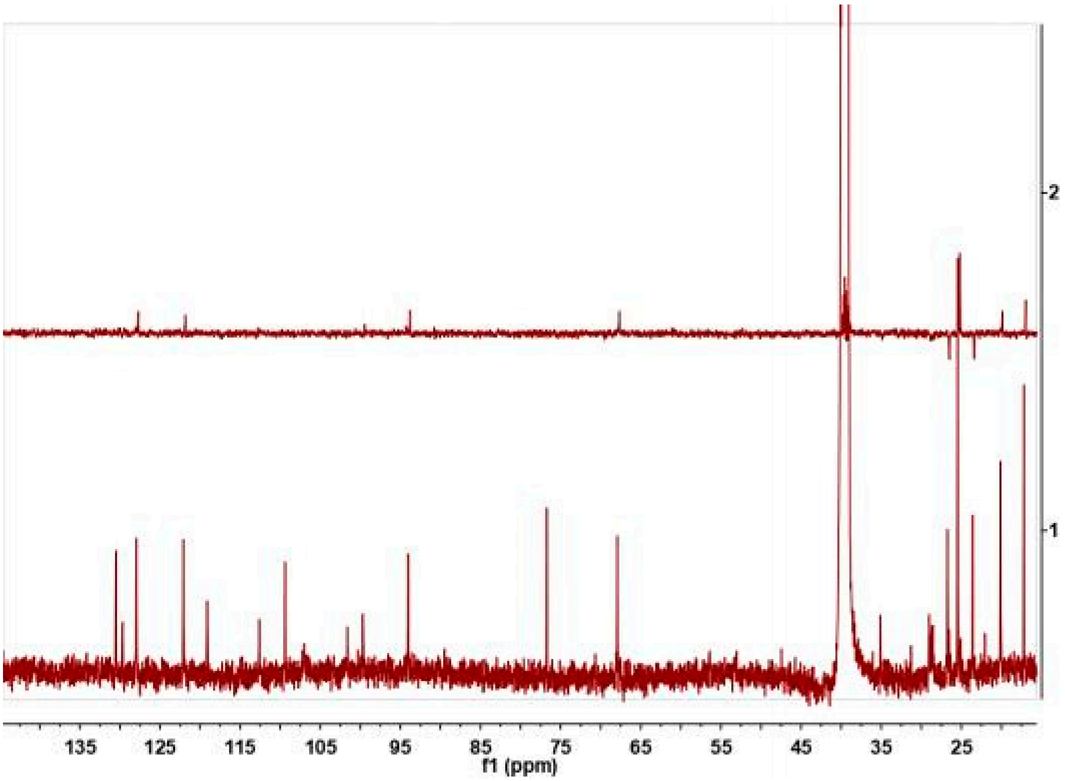

Figure S25. DEPT 135 spectrum (150 MHz, DMSO) of compound **12**.
